# Upregulated lncARAT in Schwann cells promotes axonal regeneration by recruiting and activating proregenerative macrophages

**DOI:** 10.1186/s10020-022-00501-9

**Published:** 2022-06-29

**Authors:** Gang Yin, Yaofa Lin, Peilin Wang, Jun Zhou, Haodong Lin

**Affiliations:** Department of Orthopedic Surgery, Shanghai General Hospital, Shanghai Jiao Tong University School of Medicine, Haining Road100, Shanghai, 200080 People’s Republic of China

**Keywords:** Schwann cells, Macrophages, Exosomes, LncARAT, Peripheral nerve repair

## Abstract

**Background:**

Axonal regeneration following peripheral nerve injury (PNI) depends on the complex interaction between Schwann cells (SCs) and macrophages, but the mechanisms underlying macrophage recruitment and activation in axonal regeneration remain unclear.

**Methods:**

RNA sequencing (RNA-seq) was conducted to identify differentially expressed long noncoding RNAs (DElncRNAs) between crushed sciatic nerves and intact contralateral nerves. The putative role of lncRNAs in nerve regeneration was analyzed in vitro and in vivo.

**Results:**

An lncRNA, called axon regeneration-associated transcript (lncARAT), was upregulated in SCs and SC-derived exosomes (SCs-Exo) after sciatic nerve injury. LncARAT contributed to axonal regeneration and improved motor function recovery. Mechanistically, lncARAT epigenetically activated C–C motif ligand 2 (CCL2) expression by recruiting KMT2A to CCL2 promoter, resulting in increased histone 3 lysine 4 trimethylation (H3K4me3) and CCL2 transcription in SCs. CCL2 facilitated the infiltration of macrophages into the injured nerves. Meanwhile, lncARAT-enriched exosomes were released from SCs and incorporated into macrophages. LncARAT functioned as an endogenous sponge to adsorb miRNA-329-5p in macrophages, resulting in increased suppressor of cytokine signaling (SOCS) 2 expression, which induced a proregenerative function of macrophages through a signal transducer and activator of transcription (STAT) 1/6-dependent pathway.

**Conclusions:**

LncARAT may represent a promising therapeutic avenue for peripheral nerve repair.

**Supplementary Information:**

The online version contains supplementary material available at 10.1186/s10020-022-00501-9.

## Introduction

Unlike the central nervous system (CNS), the peripheral nervous system (PNS) has higher regenerative abilities following mechanical injury and toxic or inflammatory responses (Mahar and Cavalli 2018; Lopez-Verrilli et al. 2013). The PNS possesses intrinsic regenerative capacity primarily due to SCs, which are the glia cells that form and maintain the myelin sheath around axons and support axonal regeneration (Scheib and Hoke 2013; Jessen and Mirsky 2016). Successful regeneration depends upon both neurons and nonneuronal cells, such as SCs and immune cells (Chen et al. 2015a). Inflammatory responses are primarily mediated by macrophages in response to PNI. Macrophages are highly heterogeneous and can be polarized to a proinflammatory phenotype (CD86+) or an anti-inflammatory phenotype (CD206+) by their microenvironment (Brown et al. 2012; Gordon and Taylor 2005). Circulating macrophages are attracted to the lesion site after injury, whereby they not only exert a crucial role in engulfing the inhibitory regeneration signals from myelin debris but also activate their proregenerative functions to facilitate axon regeneration (Hikawa and Takenaka 1996; Zigmond and Echevarria 2019; Tomlinson et al. 2018).

Emerging studies have demonstrated that the interactions between SCs and macrophages exert a crucial role in Wallerian degeneration and axonal regeneration in response to PNI (Stratton et al. 2018). First, SCs contribute to macrophage infiltration by secreting various ligands, including cytokines and chemokines. SC-derived gelsolin, an actin filament-severing protein, is essential for macrophage motility and macrophage recruitment to injury sites in vivo (Goncalves et al. 2010). SC-secreted periostin facilitates macrophage infiltration and results in autoimmune peripheral polyneuropathy (Allard et al. 2018). Several chemokines and cytokines, such as CCL2, interleukin (IL)-1α, IL-1β and tumor necrosis factor (TNF)-α, are also produced by SCs to promote macrophage recruitment (Martini et al. 2008; Karanth et al. 2006; George et al. 1999; Shamash et al. 2002). Second, macrophages regulate the maturation of SCs after PNI. Growth arrest-specific 6 (Gas6) is expressed by macrophages to regulate SC function, and loss of Gas6 in macrophages causes an abnormal SC response and impairs SC remyelination (Stratton et al. 2018). Microvesicles derived from CD206+ macrophages contribute to the proliferation and migration of SCs (Zhan et al. 2015). However, the mechanisms by which SCs regulate macrophage function remain poorly understood.

Exosomes play an important role in cell–cell communication by transferring biological cargo, including protein, RNA and DNA, between different cells (Lopez-Verrilli et al. 2013). Recent studies have demonstrated that SCs-derived exosomes (SCs-Exo) supports axonal regeneration (Lopez-Verrilli et al. 2013). Furthermore, modification of the exosomal miRNA profile facilitates neurite growth following nerve injury (Lopez-Leal et al. 2020). LncRNAs, a large class of non-coding transcripts greater than 200 nt in length, have emerged as key regulators of axonal regeneration and functional recovery after nerve injury (Yao et al. 2020; Wang et al. 2019). However, the regulatory roles of the lncRNA/miRNA/mRNA axis in these processes remain unclear. In the present study, we identified a novel lncRNA, NONRATT026656.2, termed axon regeneration-associated transcript (lncARAT), which was upregulated in SCs in response to injury. Upregulated lncARAT promoted CCL2 expression to recruit macrophages to injured nerves. Furthermore, lncARAT was transferred into macrophages via SCs-Exo and induced a proregenerative activation/phenotype.

## Materials and methods

### Rat model of sciatic nerve crush injury

All experimental animal protocols were approved by the Ethics Committee of the Shanghai General Hospital, Shanghai JiaoTong University School of Medicine. Adult male Sprague-Dawley rats (180–220 g) were obtained from the Zhejiang Chinese Medical University Laboratory Animal Research Center (Zhejiang, China) and maintained in 12-h light/12-h dark conditions with ad libitum access to food and water. Every effort was made to minimize the number of animals used and their suffering. The rat model of sciatic nerve crush injury was established according to a previous description (Dadon-Nachum et al. 2012). In brief, rats were anesthetized with 1% pentobarbital sodium (40 mg/kg, intraperitoneally), fixed on the operating table and disinfected with 1% iodophor solution. An upper right femoral posterior incision was made, skin and subcutaneous fascia were incised layer by layer, and the sciatic nerve was fully exposed. At a distance of 10 mm above the bifurcation into the tibial and common fibular nerves, the left sciatic nerve was crushed using fine forceps for 30 s (54 N compressive force).

### RNA sequencing

The proximal stumps of crushed sciatic nerves (0.5 cm) and intact contralateral nerves were collected 4 days postinjury (dpi). Total RNA was isolated using TRIzol reagent (Sigma-Aldrich, St. Louis, MO, USA), treated with DNase Ι (Invitrogen, Carlsbad, CA, USA) at 37 °C for 60 min, subjected to RiboMinus Eukaryote Kit (Invitrogen) to remove rRNA, and then treated with RNase R at 37 °C for 180 min. Next, RNA-seq libraries were prepared using the Illumina TruSeq RNA Sequencing protocol and subjected to RNA-seq on an Illumina HiSeq 2000 at Shanghai Biotechnology Corporation.

### Bioinformatics analysis

For filtering DElncRNAs and DEmRNAs, log 2 |FC| > 1 and p < 0.001 were used for subsequent analysis. LncRNA–mRNA coexpression analysis was performed using Cytoscape v3.4.0 (Cytoscape Consortium, CA, USA) to assess differentially expressed (DE) lncRNAs and DEmRNAs, as previously described (Ma et al. 2018). A Pearson coefficient ≥ 0.99 was used as the criterion to verify coexpression relationships.

Gene Ontology (GO) enrichment analysis (three categories: biological process, cellular component, and molecular function) was conducted using the DAVID online tool (https://david.ncifcrf.gov/) as previously described (Huang et al. 2009). p < 0.05 was set as the cutoff criteria.

### Electrophysiological assessment

At 28 dpi, rats were subjected to an electrophysiological test according to previously described protocols (n = 5) (Wang et al. 2014). In brief, the sciatic nerve near the repair site was re-exposed, a pair of stimulating electrodes (13 mm long, 0.5 mm in diameter) was inserted 3 mm near the crushed site to stimulate the sciatic nerve, and a pair of needle electrodes was subcutaneously inserted into the middle of the intrinsic foot muscle to record the compound muscle action potential (CMAP) using an EMG evoked potentiometer (MEB-9200K, Nihon Kohden, Japan). The amplitude and latency of each test were analyzed to determine the nerve conduction intensity and nerve conduction velocity, respectively.

### Walking track analysis

To functionally analyze movement, we applied propylene pigments to the plantar surface of the hind paws of rats (28 dpi) and allowed them to walk along white paper-covered corridors. Footprints from ipsi- and contralateral paws were analyzed by measuring the print length (PL) and the distance between the 1st and 5th toes. The three parameters were combined to obtain the Sciatic Functional Index (SFI) (Navarro 2016), which quantifies changes in the walking pattern (0 for uninjured; − 100 for maximally impaired gait).

### Cell culture

Primary SCs were obtained from 4 p7 SD rats (female and male), as previously described (Campana et al. 1998). After euthanasia through an intravenous overdose of sodium pentobarbital, sciatic nerves were isolated, sterilized, finely minced, and incubated in collagenase (2 mg/mL, Sigma-Aldrich, MO, USA) and DNase (Takara) for 40 min at 37 °C. Primary SC culture was maintained in DMEM containing 10% FBS at 37 °C and 5% CO_2_ in a humidified incubator. SC cultures were passaged no more than 5 times before conducting experiments. SCs (1 × 10^5^) and macrophages (1 × 10^5^) were cocultured in DMEM containing 10% FBS using Transwell inserts (BD Biosciences, CA, USA).

U937 cells were obtained from ATCC (CRL-1593.2, CA, USA) and maintained in DMEM containing 10% FBS and 12-*o*-tetradecanoyl-phorbol-13-acetate (TPA, 10 nM, Sigma-Aldrich). Primary bone marrow-derived macrophages (BMDMs) were isolated as previously described (Chen et al. 2015b) and cultured in DMEM containing 10% FBS and 1% penicillin/streptomycin. All cells were maintained in a humidified incubator at 37 °C and 5% CO_2_.

### Isolation and identification of SCs-derived exosomes (SCs-Exo)

Exosomes were isolated from SCs using Exoquick Reagent (SBI) according to the manufacturer’s instructions. Briefly, conditioned media were incubated with Exoquick reagent (5:1) for at least 12 h and then centrifuged at 1500*g* for 30 min. The pelleted exosomes were then resuspended in 100 mL PBS.

Transmission electron microscopy (TEM) was used for morphological observation. In brief, exosomes were fixed in 2.5% glutaraldehyde overnight at 4 °C. The solution was subsequently centrifuged at 100,000×*g* to remove glutaraldehyde, and exosomes were washed three times with PBS. Then, exosomes were stained with 3% phosphotungstic acid aqueous solution, fixed on copper mesh formvar grids, and examined by TEM (JEM-1010; JEOL, Tokyo, Japan).

Moreover, a partial sample of exosomes without dilution was used to analyze the distribution of exosome particle size with ZetaView^®^ Nanoparticle-tracking analysis (NTA) equipment (Particle Metrix, Meerbusch, Germany) (Capello et al. 2019). Ten micrograms of exosomes (resuspended in PBS) were used to treat macrophages according to previous reports (Lopez-Verrilli et al. 2013) and our preliminary results.

### Exosome labeling and tracking

Exosomes were isolated from culture medium and labeled with PKH67 green fluorescent membrane linker dye (Sigma-Aldrich) according to the manufacturer’s instructions. Then, the labeled exosome pellets were resuspended and added to the unstained macrophages for exosome uptake studies. After incubation for 30 min, 2 h, or 12 h at 37 °C, cells were observed by fluorescence microscopy.

### Immunofluorescence (IF)

Rat sciatic nerves were fixed in situ in 4% PFA for 10 min, dissected, embedded in O.C.T. Compound (Tissue Freezing Medium; Solarbio, Shanghai, China). Sciatic nerve cryosections (5-μm thick) were incubated with acetone for 10 min at − 20 °C, washed in PBS/0.1% Tween 20, blocked for 30 min at room temperature (RT) in blocking buffer (0.3% Triton X-100/10% goat serum/phosphate buffer saline ¼ PBS), and incubated with primary antibodies overnight at 4 °C in blocking buffer. Sections were then washed 3 times in blocking buffer, and sections were incubated with secondary antibodies for 1 h at RT in the dark. Sections were washed again, incubated with DAPI for 5 min at RT, washed and mounted in Citifluor (Agar Scientific).

The primary antibodies used for IF were as follows: neurofilament (1:1000, Abcam, ab8135), SCG10 (1:500, Abcam, ab115513), IBA1 (1:100, Abcam, ab178847), and CD68 (1:100, Abcam, ab125212). All secondary antibodies were also purchased from Abcam. Images were acquired using a Leica TCS SP-II confocal microscope.

### Fluorescence in situ hybridization (FISH)

The subcellular localization of lncARAT was assessed using FISH assay with RiboTM lncRNA FISH Probe Mix (Green) (RiboBio, Guangzhou, China). Sciatic nerve tissue sections were fixed in 4% PFA. Slides were pretreated with protease K (2 μg/mL), glycine and acetic anhydride, followed by prehybridization for 1 h and hybridization at 42 °C with probes (250 μL, 300 ng/mL) against lncARAT. Finally, slides were stained with PBS with DAPI (Sigma-Aldrich). Finally, five random fields acquired from each slide were observed and imaged using a fluorescence microscope.

### Electron microscopy analysis

Sciatic nerves were fixed in situ in 4% PFA and 0.15% glutaraldehyde in 0.1 M phosphate buffer (pH 7.4). Fixed tissues were postfixed in 2% osmium tetroxide, dehydrated in a graded acetone series, and embedded in Spurr’s resin (Electron Microscopy Sciences, EMS). Semithin sections were stained with 1% toluidine blue for analysis under a light microscope, and ultrathin sections (70-nm thick) were made. All analyses were performed 5 mm distal to the lesion site. No contrasting reagent was applied. Images were acquired using a Philips CM 100 BIOTWIN equipped with a Morada side mounted digital camera (Olympus).

### RNA pull-down and RNA immunoprecipitation (RIP)

RNA pull-down was performed using the Magnetic RNA-Protein Pull down Kit (Thermo Scientific) according to the manufacturer’s instructions. Biotin-labeled RNA (3 μg) and 1 mg of extract were used in each pulldown assay. The retrieved protein was separated on PAGE gels and visualized by standard immunoblotting.

The RIP assay was performed using the EZ-Magna RIP kit (Millipore, MA, USA). In brief, 1 × 10^7^ cells were harvested and lysed in RIP lysis buffer with one freeze–thaw cycle. Cell extracts were coimmunoprecipitated using anti-KMT2A (ab272023), anti-KMT2B (ab104444), anti-KMT2D (ab224156) or Ago2 (ab226943) antibody, and the retrieved RNA was subjected to qRT-PCR analysis.

### Chromatin immunoprecipitation (ChIP) and chromatin isolation by RNA purification (ChIRP) analysis

ChIP experiments were performed using a ChIP kit (Millipore, MA, USA). A total of 1 × 10^6^ cells were fixed in 1% formaldehyde at room temperature for 10 min, and the nuclei were isolated with nuclear lysis buffer supplemented with a protease inhibitor. The chromatin was sonicated and sheared to lengths between 100 and 200 bp. The sheared chromatin was immunoprecipitated at 4 °C overnight using anti-KMT2A antibody or anti-H3K4me3 antibody (Abcam, MA, USA). ChIP-qRT-PCR primers are listed in Additional file 6: Table S1.

The Magna ChIRP RNA Interactome Kit was obtained from Millipore (Millipore, MA, USA). Probes were designed using a single-molecule FISH online designer, biotin-labeled at the 3′ end, and divided into an “odd” or “even” group. A total of 2 × 10^7^ cells were cross-linked for each hybridization reaction. Then, the cell lysate was sonicated to shear the chromatin into 100–200 bp fragments. The sonicated cell lysates were hybridized in a mixture of biotinylated DNA probes for 4 h at 37 °C. Then, binding complexes were recovered using streptavidin-conjugated magnetic beads. Finally, DNA, RNA and protein were eluted and purified from the beads. The probes used in the ChIRP assay are listed in Additional file 6: Table S1.

### Overexpression and RNA interference (RNAi)

Recombinant lentiviruses harboring lncARAT (Lv-lncARAT) or SOCS2 (Lv-SOCS2) cDNA were produced by GenePharma (Shanghai, China). An unrelated shRNA with no match in the rat genomic sequence was used as a control (Lv-Cont). Small interfering RNAs (siRNAs) to specifically inhibit lncARAT (si-lncARATs), SOCS2 (si-SOCS2s) or short hairpin RNA (shRNAs) to specifically inhibit lncARAT (sh-lncARATs) were designed and produced by GenePharma (Shanghai, China) and transfected using HiPerFect Transfection Reagent (Qiagen, CA, USA).

MiR-329-5p mimics and 2′-*O*-methyl modified miR-329-5p inhibitor (anti-miR-329-5p) were purchased from GenePharma (Shanghai, China) to overexpress and inhibit miR-329-5p, respectively. MiRNA, siRNA and shRNA sequences are shown in Additional file 6: Table S1.

### Quantitative real-time PCR (qRT-PCR)

Total RNA was extracted using TRIzol reagent (Sigma-Aldrich). Reverse transcription PCR was performed using a PrimeScript RT reagent Kit (Takara, Tokyo, Japan) and random primers (Takara) with the following conditions: 37 °C for 15 min and 85 °C for 5 s. qRT-PCR was performed using SYBR Green Supermix (Invitrogen) on an Applied Biosystems 7300 real-time PCR system. Thermocycling conditions were 95 °C for 10 min, followed by 35 cycles of 95 °C for 10 s, 58 °C for 15 s and 72 °C for 20 s, and a final 72 °C for 20 min. β-actin and U6 were used as the internal controls, and relative expression was calculated using the 2^−ΔΔCT^ method. qRT-PCR analysis was performed in triplicate, and primer sequences are shown in Additional file 6: Table S1.

### Western blot analysis

Total protein was extracted from nerve tissues or macrophages using RIPA buffer (Solarbio). The concentrations of the extracted nuclear and cytoplasmic fractions were quantified using a BCA protein assay kit (Pierce). A total of 50 μg protein per sample was separated using SDS-PAGE (10%) and then transferred to a PVDF membrane prior to blocking with 5% nonfat milk in 1× TBST overnight at 4 °C. The membranes were then incubated with anti-CCL2 (1:2000; ab25124; Abcam, Cambridge, MA, USA), anti-iNOS (1:500; ab15323; Abcam), anti-Arg1 (1:1000; ab91279; Abcam), anti-CD206 (1:1000; ab125028; Abcam), anti-β-actin (1:5000; ab8226; Abcam), and anti-SOCS2 (1:1000; PA5-17219; Thermo Fisher Scientific) primary antibodies overnight at 4 °C. After washing 3 times in 1× TBST, membranes were incubated with the corresponding HRP-conjugated secondary antibody (1:5000; ab205718; Abcam) for 1 h at room temperature. The immunoreactive proteins were visualized using an enhanced chemiluminescence reaction.

### Dual‐luciferase reporter assay

Recombinant plasmids of pGL3-lncARAT-Wt, pGL3-lncARAT-Mut, pGL3-SOCS2-3ʹUTR-Wt, and pGL3-SOCS2-3ʹUTR-Mut were constructed in our lab (Additional file 6: Table S1). HEK293 cells (0.5 × 10^5^) were plated into 48-well plates and cotransfected with 50 nM miRNA-329-5p (or miRNA control), 20 ng of either pGL3-lncARAT-Wt, pGL3-lncARAT-Mut, pGL3-SOCS2-3ʹUTR-Wt, or pGL3-SOCS2-3ʹUTR-Mut, and 2 ng of pRL-TK (Promega, Madison, WI) using HiPerFect Transfection Reagent (Qiagen). pRL-TK was served as the internal control. HEK293 cells were collected and lysed 48 h after transfection, and luciferase activity was assessed using the Dual-Luciferase Reporter Assay System (Promega).

### Cell migration

Cell migration was assessed using a Transwell chambers (BD Biosciences, 8-µm pore size, 24-well). Briefly, macrophages (1 × 10^4^) suspended in 200 mL serum-free medium were seeded into the upper chamber, and 1 × 10^4^ SCs in 800 mL medium containing 10% FBS were added to the bottom chamber. After 48 h of culture, cells were stained with 0.1% crystal violet for 30 min, and nonmigrating cells were removed. Six visual fields were randomly chosen to calculate the number of migrated cells.

### Macrophage depletion

Macrophages were depleted through i.p. administration of clodronate liposomes in rats according to a previously reported method (Zhang et al. 2016). In brief, 0.8 mL clodronate liposomes (7 mg/mL) (Clodrosome, Encapsula NanoSciences, USA) were injected into rats with CSN. Control rats received i.p. administration of equal volume of PBS liposomes.

### Statistical analysis

Data are shown as the mean ± standard deviation (SD) from at least three separate experiments. The significance of differences between groups was assessed using Student’s *t* test, and multiple group comparisons were performed using one-way ANOVA followed by the Scheffé test. SPSS 20.0 statistical software was applied for statistical analyses. *p* < 0.05 was considered statistically significant. Data collection and analysis were conducted blinded to the experimental group.

## Results

### LncRNA expression profile in crushed sciatic nerves

**Fig. 1:**
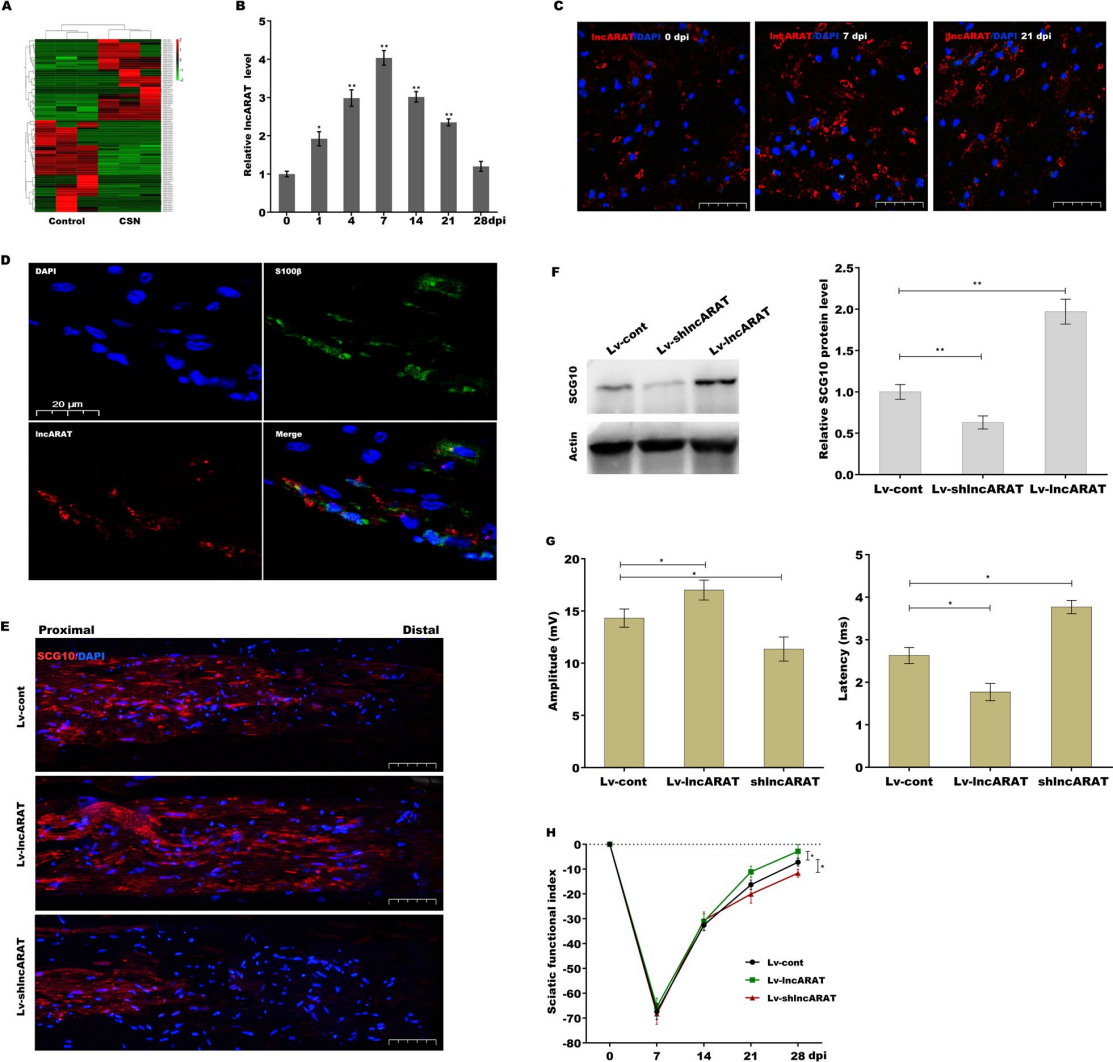
LncRNA expression profile in crushed sciatic nerves. **A** Heatmap of DElncRNAs between crushed sciatic nerves (n = 3) and intact contralateral nerves (n = 3) 4 dpi. **B** qRT-PCR analysis of lncARAT levels in crushed sciatic nerves (n = 5) at different time points postinjury (0, 1, 4, 7, 14, 21, and 28 dpi). **C** Immunofluorescence (IF) analysis of lncARAT expression in cross sections of sciatic nerves 0, 7, and 21 dpi. Scale bar, 50 µm. **D** In situ hybridization analysis of lncARAT (red) on cross sections of sciatic nerve tissues at 4 dpi. Scale bar, 20 µm. S100 β (green) was used to indicate SCs. IF (**E**) and western blot (**F**) analysis of SCG10 in injured nerves after lncARAT overexpression or knockdown at 3 dpi. Scale bar, 50 µm. **G** Quantification of latencies and amplitudes of the compound muscle action potential (CMAP) after lncARAT overexpression or knockdown (n = 5). **H** Plot of SFI obtained with walking track analysis after sciatic nerve crush in lncARAT overexpression or knockdown rats (n = 5 per group). *p < 0.05. **p < 0.01

To investigate the potential role of lncRNAs in peripheral nerve regeneration, RNA-seq was first conducted to identify DElncRNAs between crushed sciatic nerves and intact contralateral nerves. A total of 98 DElncRNAs (including 46 upregulated and 52 downregulated lncRNAs, log 2 FC > 1 and p < 0.001, Additional file 6: Table S2) were identified between crushed sciatic nerves and normal controls (Fig. 1A). GO enrichment analysis revealed that DEmRNAs (Additional file 6: Table S2, log 2 FC > 1 and p < 0.001) were enriched in 362 GO terms primarily associated with immune and cell migration (Additional file 1: Figure S1A and Additional file 6: Table S3). A Venn diagram analysis identified 4 overlapping lncRNAs ranked by *p* value and fold change (Additional file 1: Figure S1B and Additional file 6: Table S4) in accordance with a previously described method (Zhuo et al. 2019). A lncRNAs–mRNA coexpression network analysis revealed that NONRATT026656.2 (lncRNA-axon regeneration-associated transcript, hereafter called lncARAT) was correlated with multiple chemokines or chemokine receptors, including CCL2, CCR7 and CCRL2 (Additional file 1: Figure S1B), three key molecules in macrophage recruitment. Given the important role of macrophages in axonal regeneration (Hikawa and Takenaka 1996; Zigmond and Echevarria 2019; Tomlinson et al. 2018), lncARAT was selected for further investigation.

The full-length sequence of lncARAT was identified using rapid amplification of cDNA ends (RACE, Additional file 1: Figure S1C). The expected size of lncARAT was ascertained using northern blot analysis (Additional file 1: Figure S1D). Blast analysis against the NCBI database showed that rat lncARAT is located on chromosome 7, and human lncARAT is located on chromosome 12. Rat and human lncARAT overlap on the sense strand with the coding gene growth arrest specific 2 like 3 (Gas2l3). However, sequence analysis did not predict a protein greater than 60 amino acids using Open Reading Frame Finder from NCBI (Additional file 1: Figure S1E). Furthermore, we used the coding-noncoding index (CNCI), coding potential calculator algorithm (CPC2), and txCdsPredict from UCSC to calculate the coding potential of lncARAT. All three algorithms predicted that lncARAT does not possess protein-coding potential (Additional file 1: Figure S1F). Moreover, lncARAT does not contain a valid Kozak consensus sequence.

LncARAT expression was then assessed in crushed sciatic nerves at different time points postinjury. Figure 1B shows that lncARAT levels were increased 1 day postinjury (dpi), peaking 7 dpi and declining thereafter, but remained high 21 dpi compared to uninjured control nerves. Moreover, RNA-FISH assay also demonstrated that lncARAT levels were increased 7 and 21 dpi (Fig. 1C). Bifluorescence imaging analysis verified that lncARAT was primarily located in SCs (Fig. 1D). The biological function of lncARAT in nerve regeneration was investigated by injecting Lv-lncARAT or Lv-shlncARAT into sciatic nerves to enhance or repress lncARAT expression (Additional file 1: Figure S1G) and then damaging the sciatic nerves 4 days later. IF and western blot analysis revealed that the axonal regeneration marker SCG10 was markedly enhanced in response to lncARAT overexpression 3 dpi, whereas lncARAT knockdown resulted in decreased levels of SCG10 versus control nerves as shown by IF (Fig. 1E) and western blot analysis (Fig. 1F), indicating that upregulated lncARAT contributes to axonal regrowth. To evaluate the role of lncARAT in motor functional recovery after sciatic nerve damage, the amplitude and latency of the compound muscle action potential (CMAP) were assessed using electrophysiology. As shown in Fig. 1G, lncARAT overexpression resulted in significantly increased CMAP amplitude 28 dpi, whereas CMAP amplitude was decreased when lncARAT was inhibited. Moreover, lncARAT overexpression significantly decreased CMAP latency, whereas lncARAT knockdown increased CMAP latency (Fig. 1G). The walking track technique was used to further examine the role of lncARAT in motor recovery. LncARAT overexpression resulted in a significant decrease in the sciatic functional index versus controls 28 dpi, whereas lncARAT inhibition resulted in an increase in the sciatic functional index (Fig. 1H). These data demonstrate that upregulation of lncARAT promotes axonal regeneration and improves motor function recovery after sciatic nerve damage.

### LncARAT upregulates CCL2 expression in SCs

**Fig. 2:**
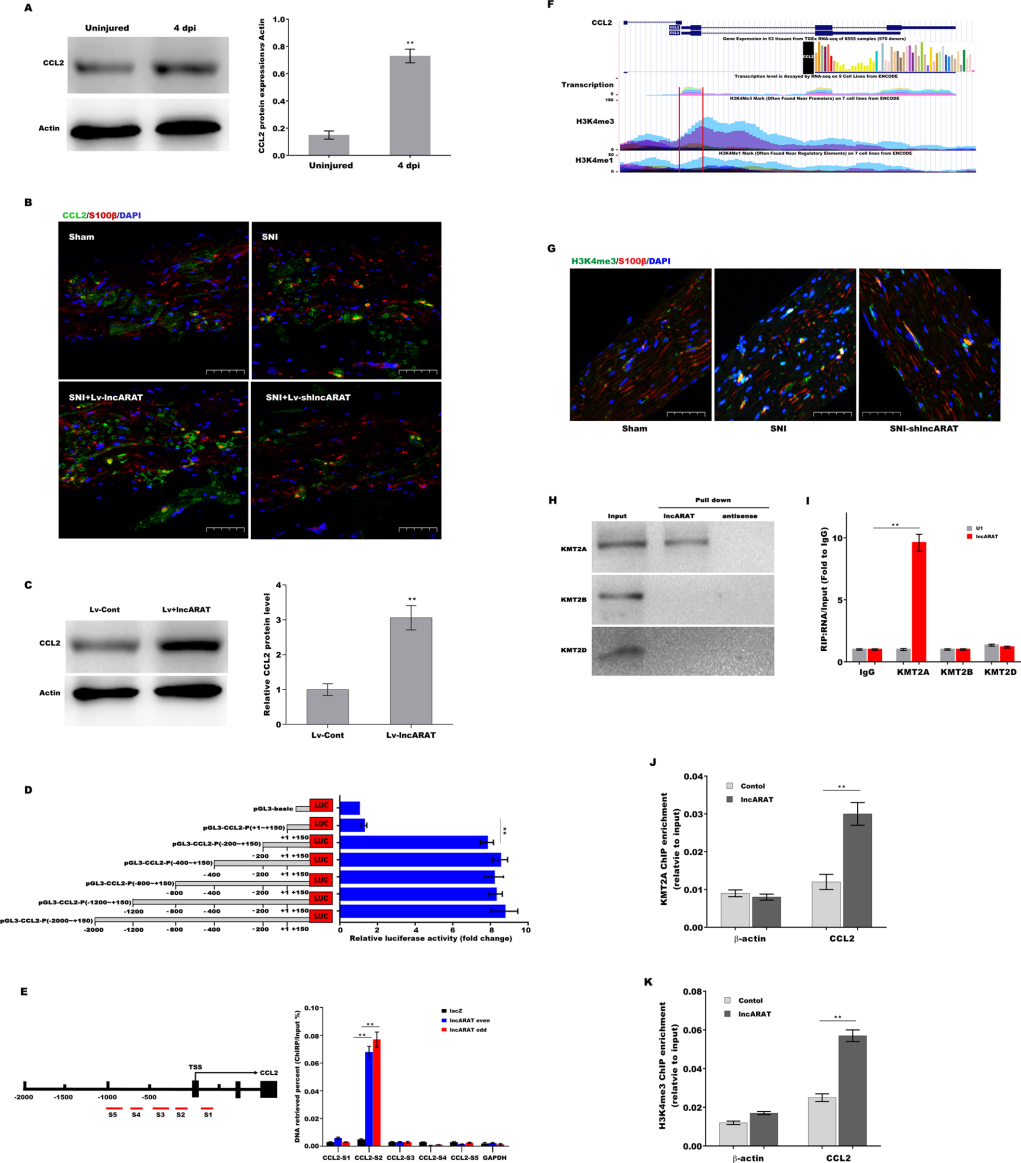
LncARAT upregulates CCL2 expression in SCs and promotes H3K4me3 at the CCL2 promoter by interacting with KMT2A. **A** Western blot and quantitative analysis of CCL2 expression in injured nerves 4 dpi (n = 5). **B** IF assay of CCL2 (green) and S100β (red) in injured nerves 4 dpi after lncARAT overexpression or knockdown. Scale bar, 50 µm. **C** Western blot and quantitative analysis of CCL2 expression in primary SCs after lncARAT overexpression for 48 h (n = 3). **D** Sequential deletions of the CCL2 promoter linked to Renilla luciferase were constructed and then transfected into SCs to assess their transcriptional activity 48 h later. **E** *Left panel,* schematic presentation of the potential lncARAT binding sites in the CCL2 promoter. *Right panel,* ChIRP assessment of lncARAT-associated chromatin in SCs. **F** Genomic neighborhood of CCL2. Genome browser tracks from the UCSC genome browser showing H3K4me3 occupancy near CCL2. **G** IF assay of H3K4me3 levels (green) and S100β levels (red) in injured nerves 4 dpi after lncARAT knockdown. Scale bar, 50 µm. **H** RNA pulldown followed by western blot analysis of KMT2A, KMT2B, and KMT2D in SCs. **I** RIP assay using anti-KMT2A, KMT2B, or KMT2D antibodies in SCs. U1 was used as the negative control. ChIP analyses using KMT2A antibody (**J**) and H3K4me3 antibody (**K**) on the regulatory regions of *CCL2* genes in SCs 72 h after lncARAT overexpression. *p < 0.05. **p < 0.01

LncARAT overexpression promoted the infiltration of macrophages into the injury site (Additional file 2: Figure S2A). Based on the results from lncRNAs–mRNA coexpression network analysis (Additional file 1: Figure S1B), we next investigated the mechanism by which lncARAT recruits macrophages. Western blot analysis showed that CCL2 was significantly upregulated in injured nerves, while CCR7 and CCRL2 were not significantly changed (Fig. 2A, Additional file 2: Figure S2B–E). Figure 2B shows that lncARAT overexpression further enhanced CCL2 expression in vivo, whereas lncARAT knockdown reduced CCL2 expression compared to the SNI group. LncARAT overexpression in primary SCs (Additional file 2: Figure S2F and G) also resulted in a significant increase in CCL2 expression (Fig. 2C). In animals in which CCL2 or CCR2 is deleted, neither macrophage infiltration nor the conditioning lesion response occurs in dorsal root ganglia (Zigmond and Echevarria 2019; Lindborg et al. 2017). Therefore, the current results indicate that SCs facilitate macrophage infiltration, at least in part, through lncARAT-induced CCL2 expression.

### LncARAT promotes H3K4me3 at the CCL2 promoter by interacting with KMT2A

To reveal the underlying mechanisms by which lncARAT upregulates CCL2 expression, a series of CCL2-luc promoter constructs were cloned, which ranged from − 2000 to + 150 nt relative to the transcriptional start site. Promoter luciferase assay exhibited a significant increase in transcriptional activity of the construct from − 200 to + 150 bp but not from + 1 to + 150 bp (Fig. 2D). Furthermore, a chromatin isolation by RNA purification (ChIRP) assay, which determines the exact locations of lncRNA binding sites on chromatin, revealed that lncARAT bound to − 19 to − 127 bp (referred to as CCL2-S2; Fig. 2E).

Integrated analysis from ENCODE tracks (http://genome.ucsc.edu/) revealed a unique chromatin H3K4me3 modification on the transcriptional edges of CCL2 (Fig. 2F). Given that many lncRNAs epigenetically regulate gene expression by regulating H3K4 methylation (Xia et al. 2017; Malek et al. 2017; Grote et al. 2013), we explored whether lncARAT enhances CCL2 expression by facilitating H3K4me3 at the CCL2 promoter. As shown in Fig. 2G, H3K4me3 levels were significantly enhanced in injured nerves, whereas lncARAT inhibition decreased H3K4me3 levels. Figure 2G also shows that H3K4me3 was clearly observed in SCs. We next performed an RNA pulldown assay to identify the histone methylation modification enzymes involved in H3K4me3 in SCs. LncARAT specifically interacted with KMT2A but not with KMT2B, KMT2D (Fig. 2H), SET1A, SET1B, or SMYD3 (Additional file 2: Figure S2H). Consistently, a RIP assay using nuclear extracts from SCs demonstrated that lncARAT directly interacted with KMT2A in SCs (Fig. 2I). To verify whether lncARAT increased CCL2 expression by interacting with KMT2A and catalyzing H3K4me3 at the CCL2 promoter, ChIP analysis was performed in SCs using KMT2A and H3K4me3 antibodies. Figure 2J and K show that lncARAT overexpression increased KMT2A occupancy and H3K4me3 levels at the CCL2 promoter. These data suggest that lncARAT upregulates CCL2 expression through KMT2A-mediated H3K4 methylation.

### SCs-Exo activates macrophages into a proregenerative phenotype

**Fig. 3:**
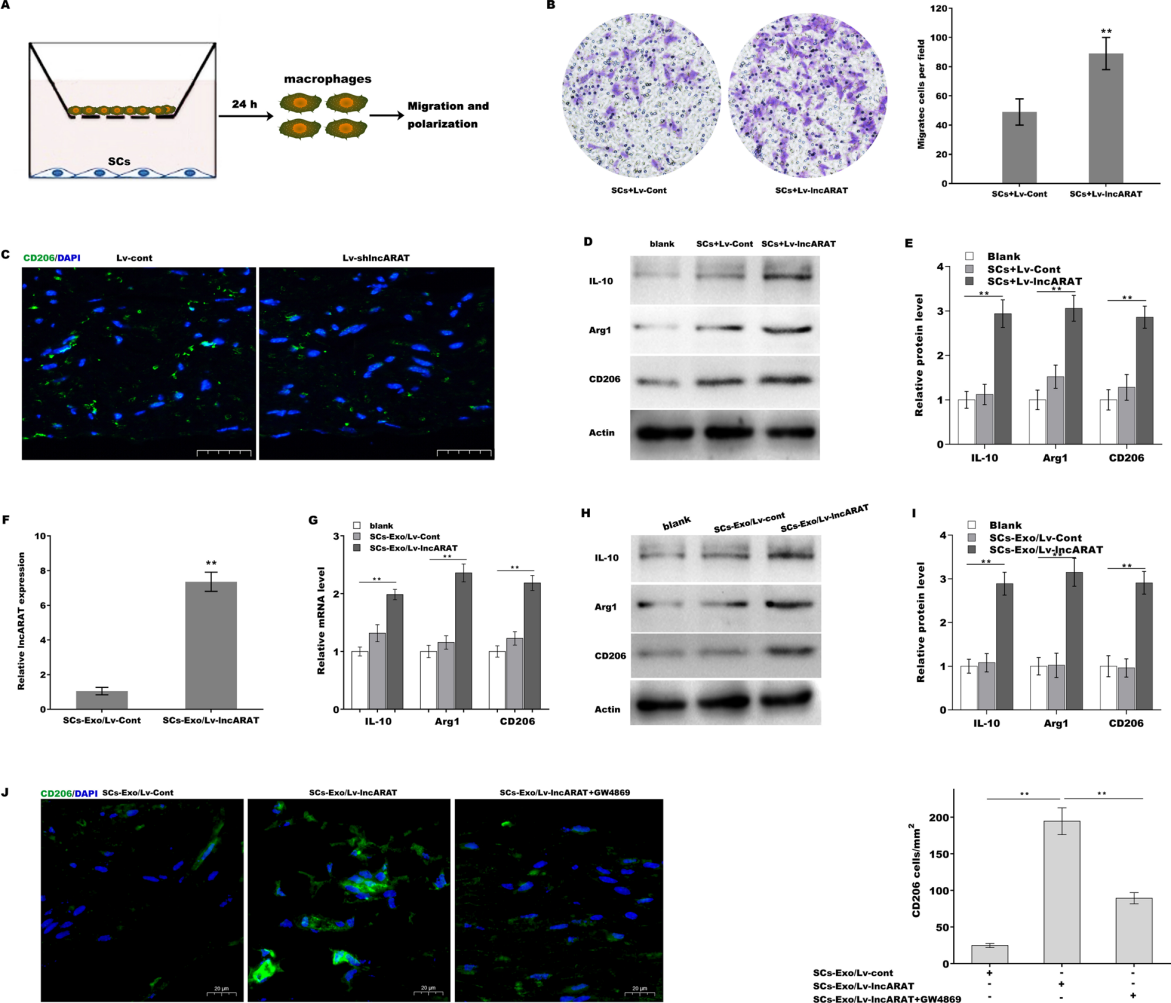
SCs-Exo activates macrophages into a proregenerative phenotype. **A** Schematic representation of an in vitro coculture system for assessing the role of SCs in regulating macrophage function. **B** Transwell migration assay of U937 cells cocultured with SCs for 48 h in the presence or absence of lncARAT overexpression. **C** IF analysis of CD206 in injured nerves 4 dpi after lncARAT knockdown. Scale bar, 50 µm. **D** and **E** Western blot and quantitative analysis of IL-10, CD206, and Arg1 expression in macrophages cocultured with SCs for 48 h in the presence or absence of lncARAT overexpression. **F** qRT-PCR analysis of lncARAT expression in SCs-Exo/cont and SCs-Exo/Lv-lncARAT. **G** qRT-PCR analysis of IL-10, CD206, and Arg1 expression in macrophages after treatment with SCs-Exo/Lv-cont or SCs-Exo/Lv-lncARAT for 48 h. **H** and **I** Western blot and quantitative analysis of IL-10, CD206, and Arg1 expression in macrophages after treatment with SCs-Exo/Lv-cont or SCs-Exo/Lv-lncARAT for 48 h. **J** IF and quantitative analysis of CD206 expression in macrophages after treatment with SCs-Exo/Lv-lncARAT in the presence or absence of GW4869 (exosome inhibitor, 10 µM). Scale bar, 50 µm. *p < 0.05. **p < 0.01

Consistent with the in vivo results, lncRNA overexpression in cultured SCs facilitated macrophage migration, including U937 (Fig. 3A and B) and BMDM migration (Additional file 3: Figure S3A and B). Given the important role of macrophage in nerve regeneration and that we found specifically CD206+ macrophages significantly increased 4 dpi (Additional file 3: Figure S3C), we sought to investigate the effect of lncARAT on regulating macrophage activation and function. IF analysis revealed that CD206+ macrophages were observed in injured nerves, whereas lncARAT knockdown dramatically decreased the number of CD206+ macrophages (Fig. 3C). An in vitro coculture system was applied to assess the association of lncARAT overexpression in SCs with macrophage activation. Macrophages cocultured with lncARAT-overexpressing SCs exhibited a proregenerative function, as evidenced by upregulated protein expression of CD206, Arg-1, and IL-10 (Fig. 3D and E).

Exosomes exert a crucial role in cell–cell communication by transferring RNA and protein between different cells (Lopez-Verrilli et al. 2013). Given that lncARAT was upregulated in SCs after nerve injury and that lncARAT overexpression in SCs produced CD206+ macrophages in in vitro coculture system, it was essential to investigate whether lncARAT was transferred from SCs to macrophages via SCs-Exo. To this end, lncARAT was overexpressed in SCs, and then the corresponding exosomes (SCs-Exo/Lv-lncARAT) were collected from the conditioned media (CM) and characterized by TEM, NanoSight and western blot analysis (Additional file 3: Figure S3D). As expected, lncARAT expression was markedly upregulated in SCs-Exo/Lv-lncARAT compared to SCs-Exo/Lv-Cont (Fig. 3F). Functionally, although SCs-Exo/Lv-lncARAT did not affect macrophage migration (Additional file 3: Figure S3E), macrophages treated with SCs-Exo/Lv-lncARAT were activated into CD206+ macrophages (Fig. 3G–I). IF analysis revealed that GW4869, an exosome inhibitor (Trajkovic et al. 2008; Takeuchi et al. 2015), markedly decreased SCs-Exo/Lv-lncARAT-induced CD206+ macrophages (Fig. 3J). Taken together, these results suggest that SCs facilitate macrophage migration by secreting CCL2 and induce a proregenerative activation via exosome-mediated SC-macrophage communication.

### LncARAT is transferred from SCs to macrophages via SCs-Exo

**Fig. 4:**
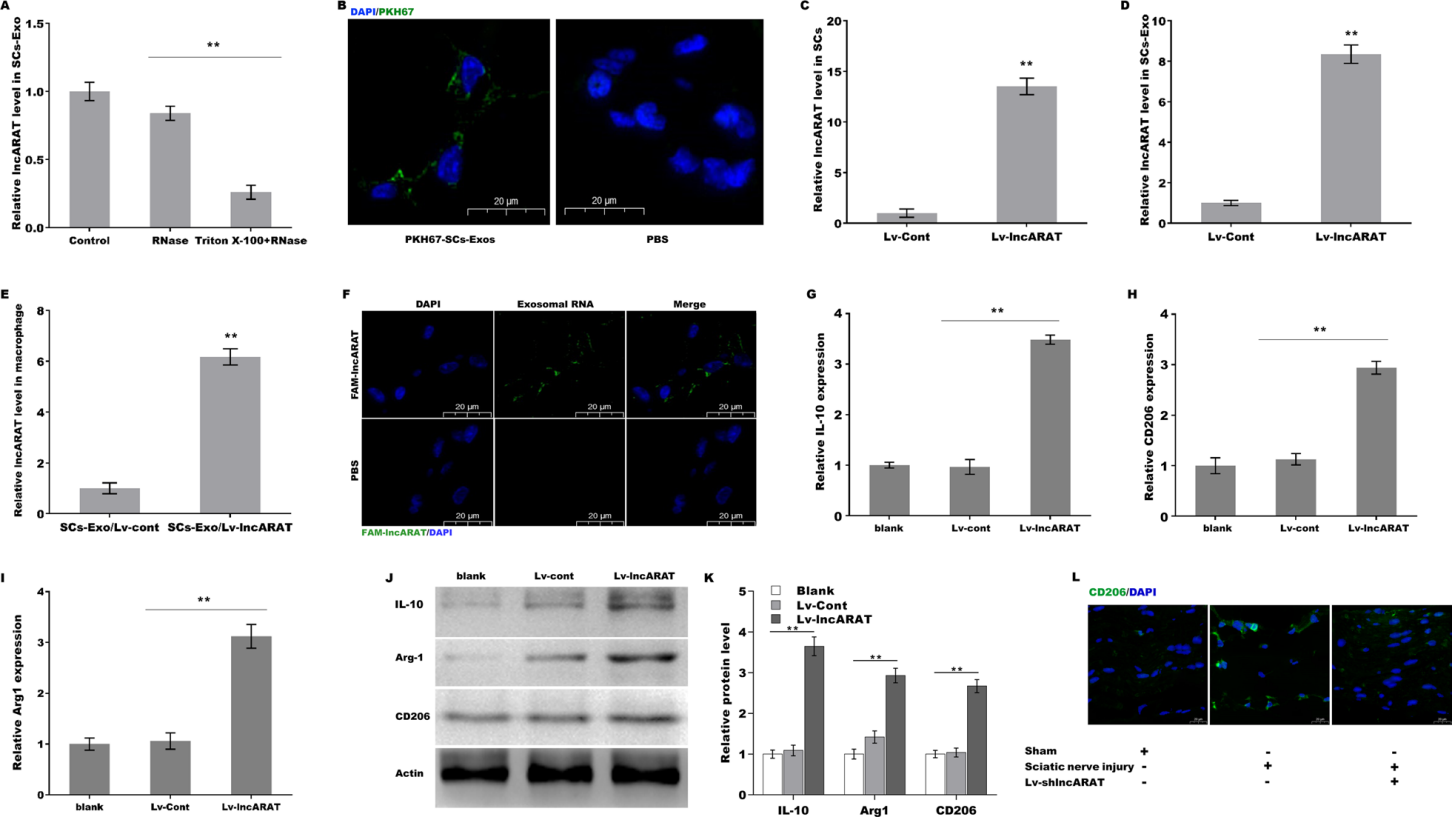
LncARAT is transferred from SCs to macrophages via SCs-Exo. **A** qRT-PCR analysis of lncARAT expression in SCs-Exo after RNase treatment in the presence or absence of Triton X-100. **B** Fluorescence microscopy analysis of U937 macrophages treated with PKH67 (green)-labeled SCs-Exo. PBS served as a negative control. Scale bar, 20 µm. qRT-PCR analysis of lncARAT expression in SCs (**C**) and SCs-Exo (**D**) after lncARAT overexpression. **E** qRT-PCR analysis of lncARAT expression in U937 macrophages treated with SCs-Exo/Lv-cont or SCs-Exo/Lv-lncARAT. **F** Fluorescence microscopy analysis of U937 macrophages treated with SCs-Exo/FAM-lncARAT. Scale bar, 20 µm. **G**–**I** qRT-PCR analysis of IL-10, CD206, and Arg1 expression in U937 macrophages after treatment with Lv-cont or Lv-lncARAT for 48 h. **J** and **K** Western blot and quantitative analysis of IL-10, CD206, and Arg1 expression in U937 macrophages after treatment with Lv-cont or Lv-lncARAT for 48 h. **L** IF assay of CD206 expression in cross sections of sciatic nerves at 4 dpi from sham rats or rats with sciatic nerve injury after Lv-lncARAT knockdown. Scale bar, 100 µm. *p < 0.05. **p < 0.01

To investigate whether lncARAT was transferred from SCs to macrophages via SCs-Exo, we first assessed whether lncARAT was packed within SCs-Exo to resist RNase-mediated degradation. Figure 4A shows that the lncARAT level in SCs-Exo remained unchanged after RNase A treatment, while additional treatment with Triton X-100 resulted in a significant decrease in lncARAT levels, indicating that lncARAT was contained within the exosomes. We then investigated whether lncARAT could be transferred from SCs to macrophages. SCs-Exo was labeled with PKH67, and then PKH67-SCs-Exo was used to treat macrophages. As shown in Fig. 4B, green fluorescence signals were observed in macrophages, indicating that SCs-Exo was internalized by macrophages. Forced expression of lncARAT in SCs resulted in a significant enhancement of lncARAT expression in SCs and SCs-Exo (Fig. 4C and D). Importantly, lncARAT levels in recipient macrophages were increased after treatment with SCs-Exo/Lv-lncARAT (Fig. 4E). LncARAT was labeled with fluorescein amidite (FAM), and SCs-Exo was acquired after FAM-lncARAT transfection to treat macrophages. Figure 4F shows that FAM-lncARAT was observed in macrophages, indicating that lncARAT was contained within SCs-Exo and transferred to macrophages.

We next investigated whether SCs-Exo activated the genes expression associated with regenerative functions of macrophages by delivering lncARAT. Forced expression of lncARAT enhanced the mRNA (Fig. 4G–I) and protein (Fig. 4J and K) expression of CD206, Arg-1, and IL-10 compared to blank and Lv-cont. In vivo, CD206 expression in crushed sciatic nerves was increased compared to that in the sham controls, whereas lncARAT depletion repressed injury-induced CD206 expression (Fig. 4L), indicating a positive correlation between lncARAT and CD206+ macrophages.

### LncARAT functions as a ceRNA and sponges miRNA-329-5p to generate proregenerative macrophages

**Fig. 5:**
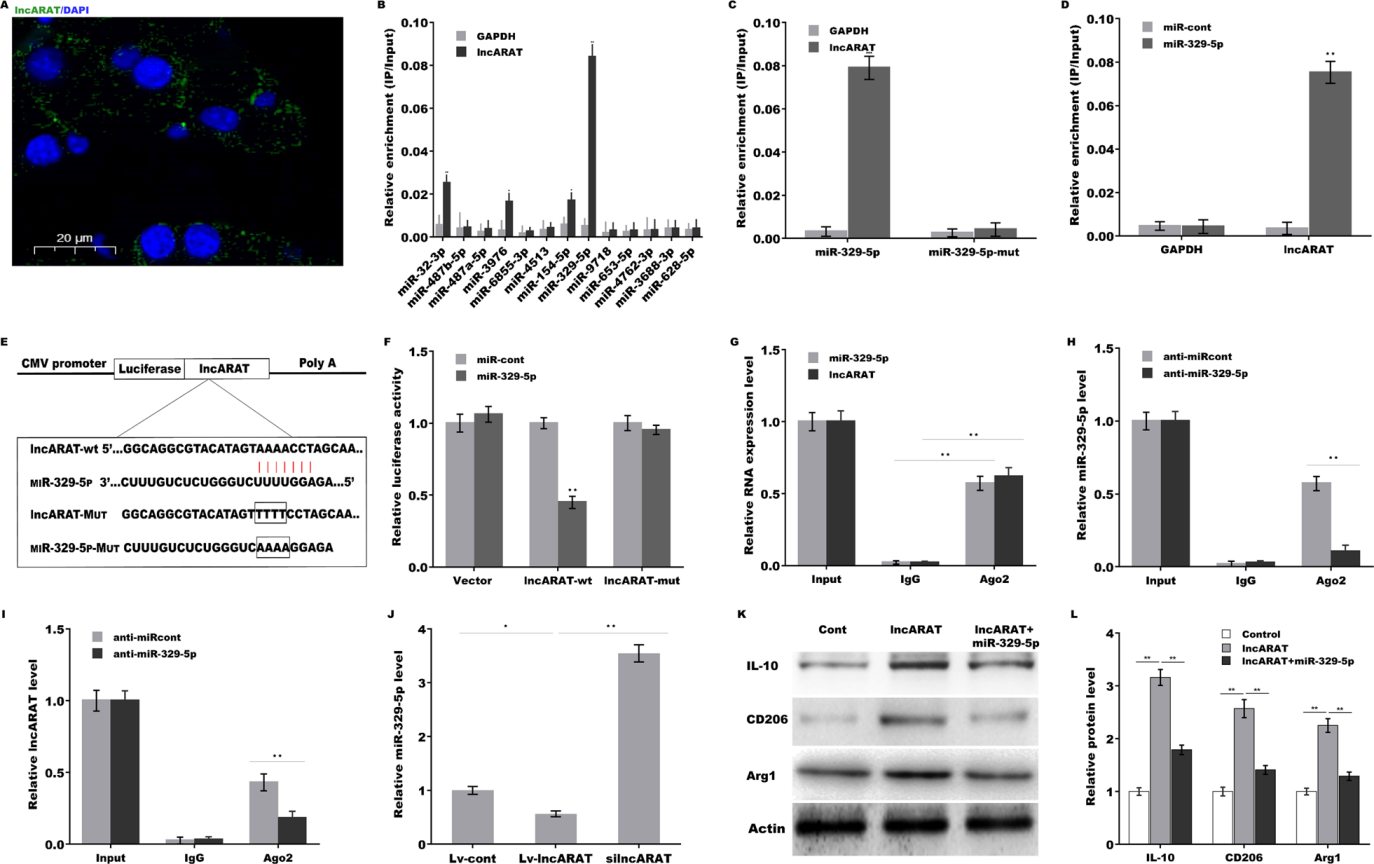
LncARAT sponges miRNA-329-5p to induce macrophages activation into a proregenerative phenotype. **A** Subcellular localization analysis of lncARAT in U937 macrophages using RNA-FISH analysis. Scale bar, 20 µm. **B** Biotinylated miRNAs were transfected into U937 macrophages, and then an RNA pull-down assay was performed to assess the interaction of lncARAT with specific miRNAs. **C** Biotinylated miRNA-329-5p mutants were transfected into U937 macrophages, and then an RNA pull-down assay was performed. **D** Biotinylated lncARAT was transfected into U937 macrophages, and then an RNA pull-down assay was performed to assess miR-329-5p enrichment. **E** Schematic representation of the miRNA-329-5p site in lncARAT. **F** Luciferase activity was assayed in HEK293 cells after cotransfection with miRNA-329-5p and luciferase reporters containing lncARAT. **G** RIP assays with anti-Ago2 antibody were performed to assess the enrichment of lncARAT and miRNA-329-5p in U937 macrophages. RIP assays with anti-Ago2 antibody were performed to assess the enrichment of miRNA-329-5p (**H**) and lncARAT (**I**) in response to miRNA-329-5p inhibition. **J** qRT-PCR analysis of miRNA-329-5p expression in U937 macrophages after lncARAT overexpression or knockdown. Western blot (**K**) and quantitative analysis of **L** IL-10, CD206, and Arg1 expression in U937 macrophages after Lv-lncARAT overexpression in the presence or absence of miRNA-329-5p. *p < 0.05. **p < 0.01

Nuclear lncRNAs regulate chromatin modification and gene expression by acting as ‘decoys’ to prevent the interaction of histone methyltransferase or deacetylase with specific genomic loci (Sun et al. 2018; Kogo et al. 2011). LncRNAs enriched in the cytoplasm typically act as competing endogenous RNAs (ceRNAs) to posttranscriptionally regulate gene expression (Salmena et al. 2011; Wang et al. 2020). The results from subcellular localization analysis revealed that lncARAT was largely localized in the cytoplasm in macrophages (Fig. 5A), indicating that lncARAT might act as a ceRNA (Thomson and Dinger 2016). A bioinformatics tool (miRDB, http://mirdb.org/) (Wang 2016) was used to predict miRNAs that potentially sponged by lncARAT, and 36 miRNAs had complementary sequences to lncARAT (Additional file 6: Table S5). We performed an RNA pull-down assay using biotinylated miRNA to identify the miRNAs sponged by lncARAT. LncARAT was more enriched in miRNA-329-5p than in other miRNAs (Fig. 5B), whereas the miRNA-329-5p mutant in the lncARAT binding site lost its capability sponged by lncARAT in macrophages (Fig. 5C). Moreover, an RNA pull-down assay using biotinylated lncARAT further verified that miRNA-329-5p was sponged by lncARAT (Fig. 5D). MiRNA-329-5p expression was also observed in infiltrating macrophages following injury (Additional file 4: Figure S4A).

Then recombinant plasmids of pGL3-lncARAT-wt and pGL3-lncARAT-Mut (Fig. 5E) were constructed and cotransfected with miRNA-329-5p into HEK293 cells, and then luciferase activity was assessed. Figure 5F shows that miRNA-329-5p markedly repressed the luciferase activity of pGL3-lncARAT-wt compared to the miR-control, whereas the lncARAT mutation in the miRNA-329-5p binding site destroyed the inhibitory effect. Ago2 is the central protein of the “miRNA-induced silencing complex (miRISC)” and exerts a critical effect on miRNA-guided translational inhibition (Liu et al. 2004, 2017; Chendrimada et al. 2005). RIP with anti-Ago2 antibody revealed that miRNA-329-5p and lncARAT were concurrently enriched in macrophages (Fig. 5G). In particular, miRNA-329-5p inhibition markedly reduced the enrichment of lncARAT and miRNA-329-5p in Ago2 precipitates (Fig. 5H and I). Forced expression of lncARAT in macrophages resulted in decreased expression of miRNA-329-5p, whereas lncARAT knockdown upregulated miRNA-329-5p expression (Fig. 5J). Functionally, lncARAT overexpression induced macrophages activation into a proregenerative phenotype, whereas miRNA-329-5p treatment partially reversed this effect (Fig. 5K and L). These data demonstrate that lncARAT activates macrophages into a proregenerative phenotype by sponging miRNA-329-5p and disrupting its function.

### MiRNA-329-5p regulates macrophage activation by targeting SOCS2-STAT1/6 signaling

**Fig. 6:**
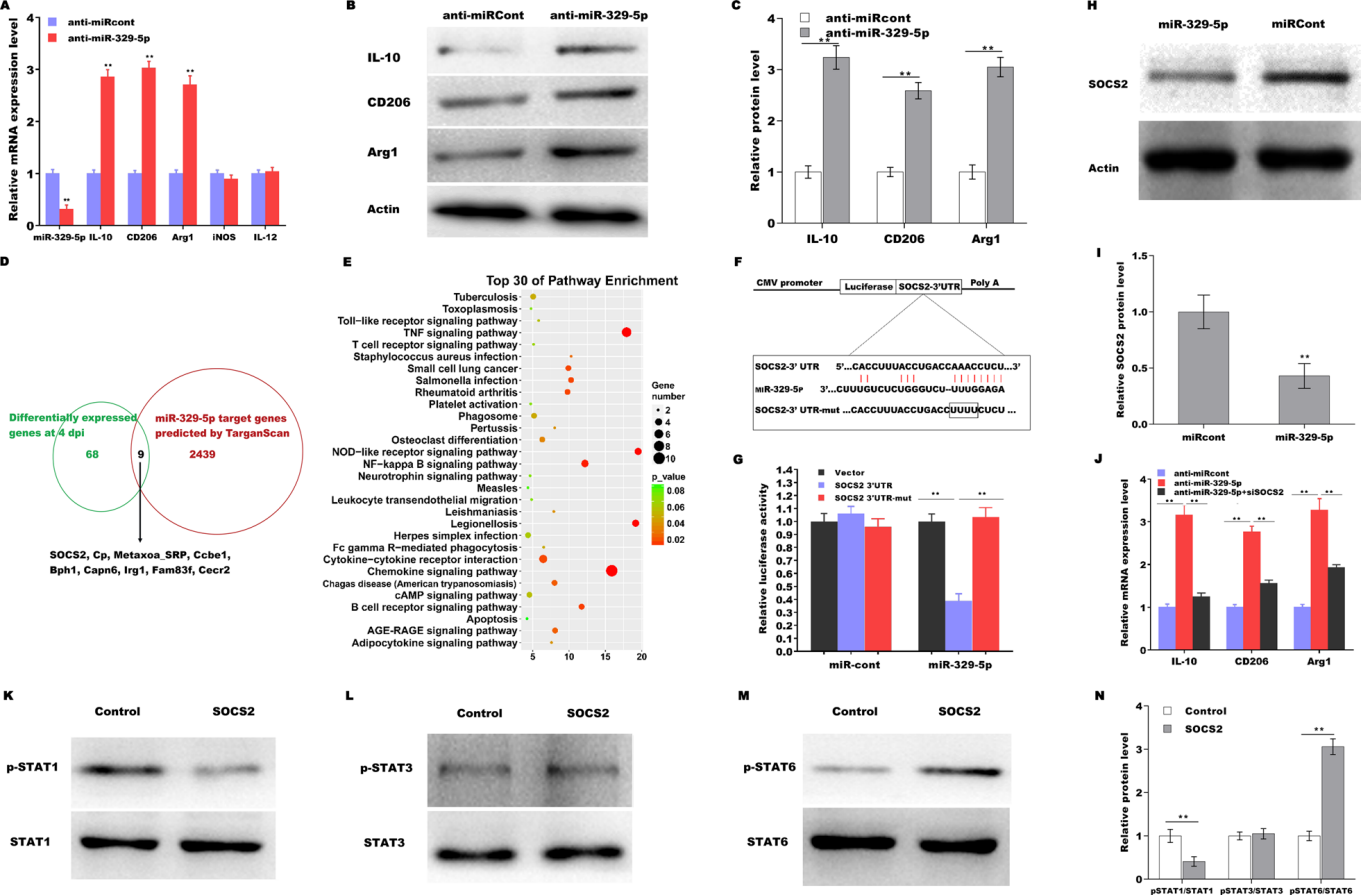
MiRNA-329-5p regulates macrophage activation by targeting SOCS2-STAT1/6 signaling. qRT-PCR (**A**) and western blot (**B** and **C**) analysis of IL-10, CD206, and Arg1 expression in U937 macrophages after miRNA-329-5p inhibition for 48 h. **D** A Venn diagram analysis of overlapping mRNAs in both sets of data. **E** KEGG analysis of DEmRNAs between crushed sciatic nerves and intact contralateral nerves. **F** Schematic representation of the miRNA-329-5p site in the SOCS2-3ʹUTR. **G** Luciferase activity was assayed in HEK293 cells after cotransfection with miRNA-329-5p and luciferase reporters containing the SOCS2-3ʹUTR. **H** and **I** Western blot and quantitative analysis of SOCS2 in U937 macrophages after miRNA-329-5p inhibition. **J** qRT-PCR analysis of IL-10, CD206, and Arg1 expression in U937 macrophages after miRNA-329-5p inhibition in the presence or absence of siSOCS2. **K**–**N** Western blot analysis of p-STAT1, p-STAT3, and p-STAT6 in U937 macrophages after SOCS2 overexpression for 48 h. *p < 0.05. **p < 0.01

The role of miRNA-329-5p in regulating macrophage polarization was next investigated. miRNA-329-5p inhibition resulted in a significant increase in the mRNA (Fig. 6A) and protein (Fig. 6B and C) levels of proregenerative markers such as IL-10, CD206, and Arg1. Bioinformatics analysis revealed that 2448 genes are potential targets of miRNA-329-5p as determine using the TargetScan tool (http://www.targetscan.org/vert_72/). Of these, 9 genes were differentially expressed in crushed sciatic nerves (Additional file 6: Table S2, Fig. 6D). KEGG analysis of DEmRNAs showed that the NF-κB and TNF signaling pathways were the primarily enriched pathways (Fig. 6E), and SOCS2, a crucial member of the NF-κB and TNF pathways, was a potential target gene of miRNA-329-5p. Then, recombinant plasmids of pGL3-SOCS2-3ʹUTR-WT (Fig. 6F) or its mutant (pGL3-SOCS2-3ʹUTR-Mut) were constructed and cotransfected with miRNA-329-5p. Figure 6G shows that miRNA-329-5p significantly inhibited the luciferase expression of SOCS2-3ʹUTR-LUC, whereas the mutation of 4 nucleotides in the 3ʹUTR of SOCS2 completely abrogated the suppressive effect. MiRNA-329-5p overexpression in macrophages further repressed the protein expression of SOCS2 (Fig. 6H and I). Functionally, miRNA-329-5p inhibition induced macrophages activation into a proregenerative phenotype, whereas SOCS2 inhibition significantly weakened the effect (Fig. 6J). Given the role of STAT signaling in macrophage function and the correlation of SOCS2 with STAT activation (Spence et al. 2013; Shen et al. 2000), we next assessed whether SOCS2 overexpression results in abnormal activation of STAT. Figure 6K–N shows that SOCS2 overexpression repressed STAT1 activation and activated STAT6 signaling but did not affect STAT3 signaling.

### The role of lncARAT/miRNA-329-5p/SOCS2 axis in generating proregenerative macrophages

**Fig. 7:**
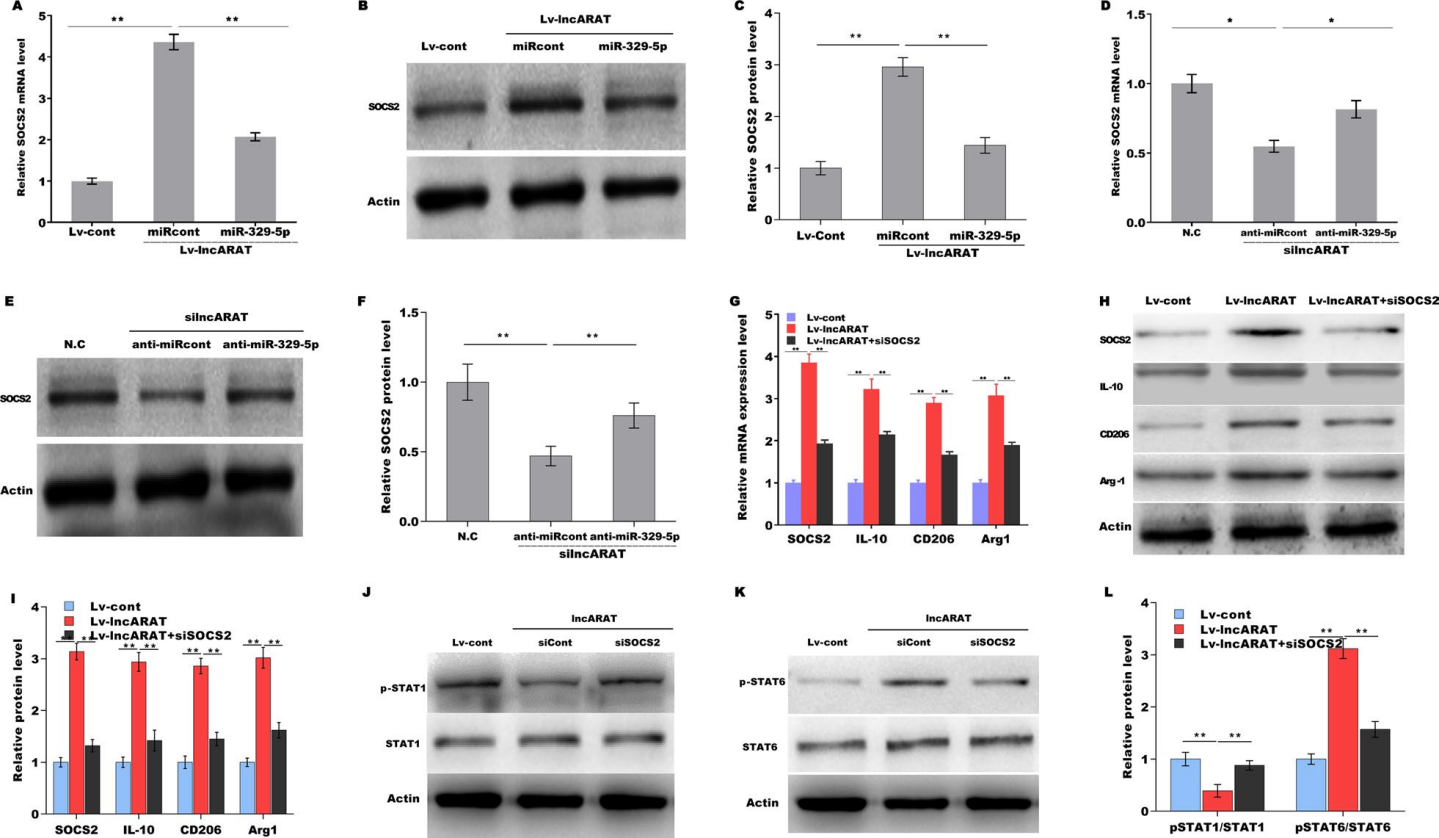
The role of the lncARAT/miRNA-329-5p/SOCS2 axis in regulating macrophage function. qRT-PCR (**A**) and western blot (**B** and **C**) analysis of SOCS2 expression in U937 macrophages overexpressing lncARAT in the presence or absence of miRNA-329-5p. qRT-PCR (**D**) and western blot (**E** and **F**) analysis of SOCS2 expression in U937 macrophages treated with silncARAT in the presence or absence of miRNA-329-5p inhibitor. qRT-PCR (**G**) and western blot (**H** and **I**) analysis of IL-10, CD206, and Arg1 expression in U937 macrophages overexpressing lncARAT in the presence or absence of siSOCS2. **J**–**L** Western blot and quantitative analysis of p-STAT1 and p-STAT6 levels in U937 macrophages overexpressing lncARAT in the presence or absence of siSOCS2. *p < 0.05. **p < 0.01

The interaction among lncARAT, miRNA-329-5p and SOCS2 and lncARAT’s role in regulating macrophage function were next investigated. Figure 7A–C shows that lncARAT overexpression upregulated the mRNA and protein levels of SOCS2 in macrophages, whereas miRNA-329-5p treatment partially repressed this effect. Moreover, lncARAT knockdown downregulated SOCS2 expression, whereas miRNA-329-5p inhibition significantly weakened this effect (Fig. 7D–F). To determine whether the lncARAT/miRNA-329-5p/SOCS2 axis regulates macrophage function, macrophages were cotransfected with lncARAT with or without siSOCS2 (Additional file 4: Figure S4B). LncARAT induced macrophages activation into a proregenerative phenotype (Fig. 7G–I), whereas SOCS2 inhibition partially repressed this effect. Based on these data, we confirmed that lncARAT acts as a ceRNA to induce a proregenerative macrophages via miRNA-329-5p/SOCS2 axis. Furthermore, lncARAT overexpression repressed STAT1 signaling activation and promoted STAT6 signaling activation, whereas SOCS2 depletion significantly weakened this effect (Fig. 7J–L).

### LncARAT facilitates axonal regeneration and functional recovery by generating proregenerative macrophages

An in vivo macrophage-exhausted model was established by injecting clodronate in the presence of Lv-lncARAT and then damaging the sciatic nerves 4 days later. As shown in Additional file 5: Figure S5A, although lncARAT overexpression promoted macrophage infiltration into injured nerves, clodronate treatment markedly depleted macrophages in the injured nerves. Consequently, the function of lncARAT in accelerating early demyelination was also damaged by clodronate (Additional file 5: Figure S5B and C). The sciatic functional index was higher in lncARAT-overexpressing rats than in control rats, whereas the sciatic functional index was similarly decreased in control rats and lncARAT-overexpressing rats following clodronate treatment (Additional file 5: Figure S5D), suggesting that the role of lncARAT in axonal regeneration and functional recovery was abolished due to macrophage exhaustion. Taken together, these data demonstrated that upregulation of lncARAT promotes axonal regeneration by recruiting macrophages and activating their proregenerative genes expression and functions (Additional file 5: Figure S5E).

## Discussion

Revealing the underlying mechanisms of SC-macrophage interactions is a prerequisite for developing medicinal treatment strategies for peripheral nerve repair. PNI causes extensively aberrant expression of both mRNAs and noncoding RNAs (lncRNAs and miRNAs). LncRNAs can function as ceRNAs to regulate mRNA expression by absorbing miRNAs. In the current study, we demonstrated that (i) lncARAT expression in SCs is increased after PNI, (ii) lncARAT promotes macrophage infiltration by increasing CCL2 expression, (iii) lncARAT promotes H3K4me3 at the CCL2 promoter by directly interacting with KMT2A, (iv) SCs-Exo activates macrophages into a proregenerative phenotype by delivering lncARAT, (v) lncARAT functions as a ceRNA and sponges miRNA-329-5p to regulate SOCS2/STAT signaling, and (vi) lncARAT-regulated macrophage function facilitates axonal regeneration and functional recovery. These data reveal the important role of lncARAT in regulating the interaction between SCs and macrophages and may serve as a promising therapeutic avenue for peripheral nerve repair.

It is well known that chemokines exert a key role in macrophage recruitment and activation after PNI. Emerging studies are exploring the underlying mechanisms of epigenetically activated CCL2 expression. A product of degenerated neural tissue, chitooligosaccharides, stimulates SCs to express CCL2 by repressing miR-327 in SCs (Zhao et al. 2017). In a cancer study, Chen et al. showed that lncRNA-LNMAT1 upregulates CCL2 expression by recruiting hnRNPL to the CCL2promoter, resulting in increased H3K4me3 to facilitate CCL2 transcription and subsequent macrophage recruitment into tumor tissues (Chen et al. 2018). In this study, we identified a novel highly expressed lncRNA in SCs after PNI, lncARAT. LncARAT overexpression enhanced expression levels of CCL2 in SCs in vivo and in vitro. Sequence analysis revealed a distinct chromatin H3K4me3 modification on the transcriptional edges of CCL2. We experimentally demonstrated that lncARAT increases CCL2 expression by recruiting KMT2A to the CCL2 promoter and results in enhanced H3K4me3, which promotes CCL2 transcription. Therefore, the current results verified that SCs recruit circulating macrophages to injured nerves through the lncARAT/KMT2A/CCL2 pathway.

The primary innovation of this study is our elucidating how SCs induce a proregenerative macrophage. Although SCs potently induce the proregenerative function of macrophage (Stratton et al. 2016), SCs cannot secrete proregenerative macrophage-associated cytokines. The underlying mechanisms by which SCs regulate macrophage function remain poorly understood. Mounting evidence has demonstrated that exosomes exert an important role in cell–cell communication (Simons and Raposo 2009; Denzer et al. 2000), and SCs-Exo contributes to axonal regeneration by communicating with neighboring axons after PNI (Lopez-Verrilli et al. 2013; Lopez-Leal and Court 2016). In this study, we further revealed the role of exosomes in the interaction of SCs with macrophages. Upregulated lncARAT was contained within SCs-Exo to resist RNase-mediated degradation and then transferred to macrophages. Subcellular localization analysis revealed that lncARAT was largely localized in the cytoplasm in macrophages, indicating that lncARAT might act as a ceRNA that regulates mRNA transcription by sponging miRNAs (Thomson and Dinger 2016). Indeed, the results from RNA pull-down and RIP assays verified the direct combination of lncARAT with miRNA-329-5p. A luciferase reporter assay demonstrated that miRNA-329-5p specifically binds to lncARAT and represses the activity of lncARAT-LUC. RNA RIP assay with anti-Ago2 antibody showed that miRNA-329-5p inhibition markedly reduces the enrichment of lncARAT and miRNA-329-5p in Ago2 precipitates, confirming the direct combination of lncARAT with miRNA-329-5p. Functionally, miRNA-329-5p inhibition activates macrophages into a proregenerative phenotype.

Then, the target gene of miRNA-329-5p was identified, and the data showed that miRNA-329-5p inhibits SOCS2 expression in macrophages. LncARAT may function as an endogenous sponge to adsorb miRNA-329-5p, resulting in increased SOCS2 expression, which induces macrophages activation through a STAT1/6-dependent pathway, promoting axonal regeneration. Taken together, the present data verify that lncARAT is an important mediator of SCs-mediated macrophage infiltration and activation that supports axonal regeneration and may serve as a promising therapeutic avenue for peripheral nerve repair.

The major limitations of the present study are as follows: (i) lncARAT-deficient (lncARAT^−/−^) rats were not successfully constructed. The role of lncARAT in recruiting macrophages and inducing CD206+ macrophages will be further verified in vivo in lncARAT^−/−^ rats. We only used lv-shlncARAT to repress lncARAT expression in injured nerves. (ii) Although the in vitro role of lncARAT in facilitating macrophage recruitment and proregenerative activation was marked, the role of lncARAT in motor recovery was not striking compared with clodronate. This may be because endogenous lncARAT is upregulated after sciatic nerve damage, and thus forced expression of lncARAT did not induce striking motor recovery.

## Conclusion

Upregulated lncARAT in SCs promotes axonal regeneration by recruiting and activating proregenerative macrophages.

## Supplementary Information

**Additional file 1: Figure S1. **LncARAT identification. (A) A total of 362 GO terms were identified by GO enrichment analysis using DEmRNAs, and these terms were mainly associated with immune and cell migration. (B) Venn diagram analysis identified 4 overlapping lncRNAs ranked by *p* value and fold change (FC), and lncRNA–mRNA co-expression network analysis showed that lncARAT was correlated with multiple chemokines or chemokine receptors including CCL2, CCR7 and CCRL2. (C) The full-length sequence of lncARAT was identified using RACE. (D) Expected size of lncARAT was ascertained using northern blot analysis. (E) The coding potential of lncARAT was analyzed using Open Reading Frame Finder from NCBI. (F) Coding-Non-Coding Index (CNCI), Coding Potential Calculator algorithm (CPC2), and txCdsPredict from UCSC were used to calculate the coding potential of lncARAT. The results from all three algorithms showed that lncARAT does not possess protein-coding potential. (G) qRT-PCR analysis was carried out to assess lncARAT expression after injecting Lv-lncARAT or Lv-shlncARAT into sciatic nerves at 4 and 7 dpi. **p < 0.01.

**Additional file 2: Figure S2. **LncARAT promoted infiltration of macrophages into injury site. (A) Immunofluorescence assay of CD68 (red) and DAPI (blue) in injured nerves after lncARAT overexpression or knockdown. Scale bar, 50 µm. (B) qRT-PCR analysis of CCR7 expression in injured nerves at 4 dpi. Western blot (C) and quantitative (D) analysis of CCR7 expression in injured nerves at 4 dpi. (E) Western blot analysis of CCRL12 expression in injured nerves at 4 dpi. qRT-PCR analysis was carried out to assess lncARAT expression after treatment with Lv-lncARAT (F) or Lv-shlncARAT (G) in primary SCs. (H) RNA pull down assay followed by western blot analysis of SET1A, SET1B, and SMYD3.***p < 0.001.

**Additional file 3: Figure S3. **SCs-Exo promoted macrophage M2 polarization. (A) Schematic presentation of an in vitro co-culture system for assessing the role of SCs in regulating BMDM migration and polarization. (B) Transwell migration assay of BMDM co-cultured with SCs with or without lncARAT overexpression. (C) qRT-PCR analysis was carried out to assess the expression of macrophage activation markers in crushed sciatic nerves (CSN) at 1 dpi and 4 dpi. (D) Exosomes (indicated by red arrows) were isolated from SCs-Exo/Lv-cont and SCs-Exo/Lv-lncARAT and analyzed through TEM, ZetaView^®^ Nanoparticle-tracking analysis (NTA) equipment, and western blot analysis of exosomal markers (CD9, CD81, and CD63). Scale bar, 500 nm. (E) Schematic presentation for assessing BMDM migration. In brief, SCs were treated with Lv-lncARAT to overexpress lncARAT and then SCs-Exo/Lv-lncARAT was collected. Transwell migration assay was carried out to assess BMDM migration after SCs-Exo/Lv-lncARAT treatment. ns, no significant.

**Additional file 4: Figure S4. **lncARAT upregulated SOCS2 expression. (A) Immunofluorescence analysis of CD68 (red) and miRNA-329-5p (green) to assess miRNA-329-5p expression in infiltrating macrophages following injury. Scale bar, 50 µm. (B) Western blot and quantitative analysis of SOCS2 expression in U937 cell after lncARAT overexpression in the presence or absence of siSOCS2. **p < 0.01.

**Additional file 5: Figure S5. **lncARAT facilitated axonal regeneration and functional recovery through interacting with macrophages. (A) An in vivo macrophage-exhausted model was established by injecting clodronate in the presence of Lv-lncARAT and damaged the sciatic nerves after 4 days, and then immunofluorescence analysis of CD68 (red) and DAPI (blue) in injured nerves was carried out to assess macrophage infiltration. Scale bar, 50 µm. (B and C) An in vivo macrophage-exhausted model was established by injecting clodronate in the presence of Lv-lncARAT and damaged the sciatic nerves after 4 days, and then electron micrographs showed the function of lncARAT and clodronate on accelerating early demyelination. Scale bar, 2 µm. (D) Plot of SFI obtained with walking track analysis after sciatic nerve crush after lncARAT overexpression in the presence or absence of clodronate (n = 5 per group). (E) Schematic presentation of the interaction between SCs and macrophages in axonal regeneration. In brief, lncRNA expression was increased in SCs and SCs-derived exosomes after crushed sciatic nerves. Upregulated lncARAT epigenetically activated CCL2 expression by recruiting KMT2A to CCL2 promoter. CCL2 facilitated the infiltration of macrophages into the injured nerves. Meanwhile, lncARAT-enriched exosomes were released from SCs and incorporated into macrophages. LncARAT functioned as an endogenous sponge to adsorb miRNA-329-5p in macrophage, resulting in an increased SOCS 2 expression, which facilitated macrophage M2 polarization through a STAT 1/6-dependent pathway, thus promoted axonal regeneration.

**Additional file 6: Table S1.** Sequence of primers, miRNA, and siRNA used in the study. **Table S2.** DElncRNAs in crushed sciatic nerve compared with intact contralateral nerve through RNA-seq analysis. **Table S3.** GO analysis of differentially expressed mRNAs. **Table S4.** The top10 lncRNAs ranked by fold change. **Table S5.** miRNA prediction sponged by lncARAT using miRDB tool (http://mirdb.org/).

## Data Availability

The data supporting the findings of the article are available in and in the GEO Dataset at https://www.ncbi.nlm.nih.gov/geo/ under reference number GSE149657. More data supporting this study are available from the corresponding author.

## Abbreviations

SCs: Schwann cells
CSN: Crushed sciatic nerves
PNS: Peripheral nervous system
SD: Sprague Dawley
CMAP: Compound muscle action potential
SFI: Sciatic functional index
BMDM: Bone marrow-derived macrophages
DNS: Damaged sciatic nerves
