# Supplementary figures and images for: Upregulated lncARAT in Schwann cells promotes axonal regeneration by recruiting and activating proregenerative macrophages
Source: Mol Med. 2022 Jun 29;28:76. doi: 10.1186/s10020-022-00501-9 (PMC9245276; doi:10.1186/s10020-022-00501-9)

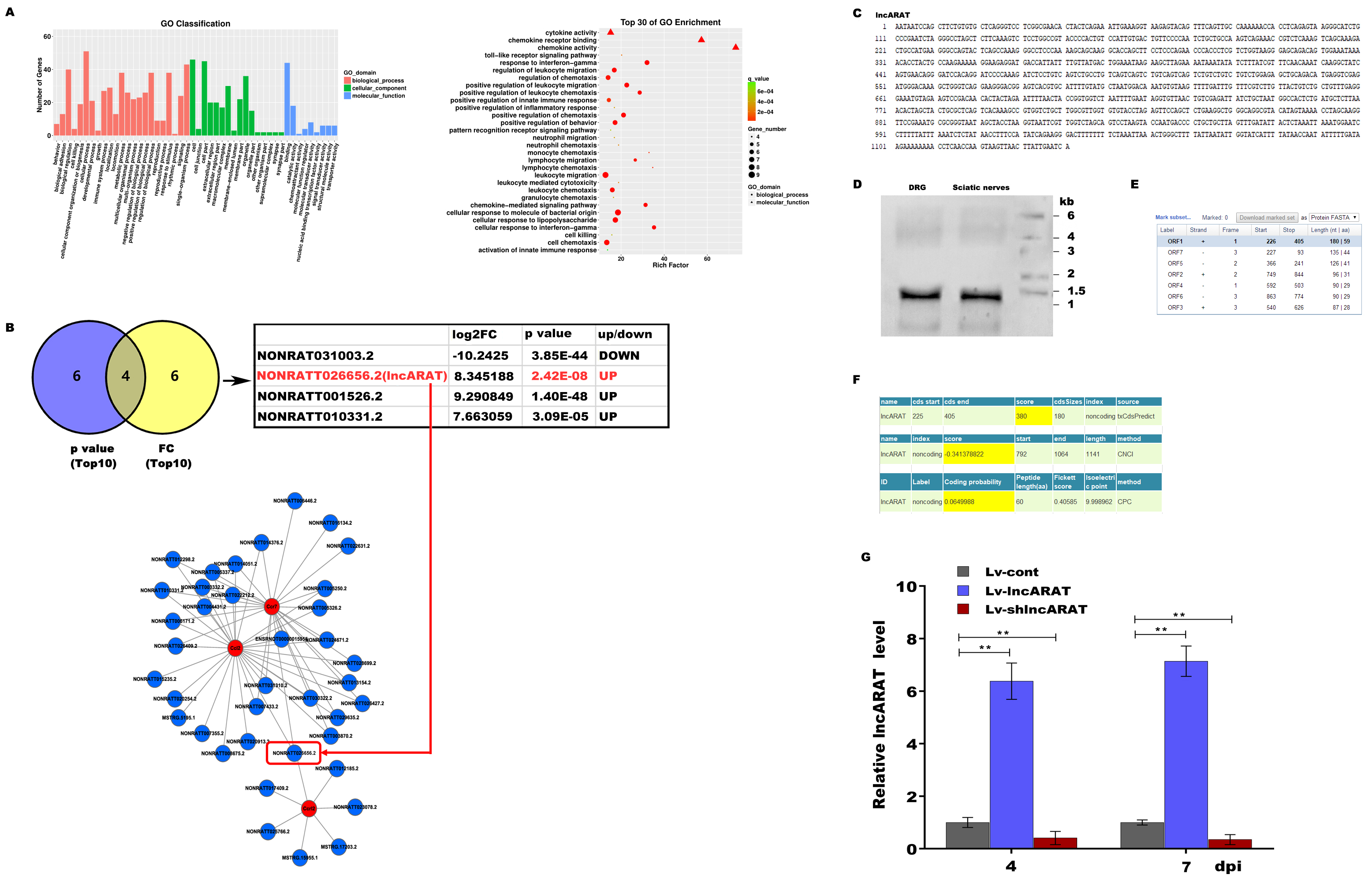

Supplement: Supplementary file 1 — Additional file 1: Figure S1. LncARAT identification. (A) A total of 362 GO terms were identified by GO enrichment analysis using DEmRNAs, and these terms were mainly associated with immune and cell migration. (B) Venn diagram analysis identified 4 overlapping lncRNAs ranked by p value and fold change (FC), and lncRNA–mRNA co-expression network analysis showed that lncARAT was correlated with multiple chemokines or chemokine receptors including CCL2, CCR7 and CCRL2. (C) The full-length sequence of lncARAT was identified using RACE. (D) Expected size of lncARAT was ascertained using northern blot analysis. (E) The coding potential of lncARAT was analyzed using Open Reading Frame Finder from NCBI. (F) Coding-Non-Coding Index (CNCI), Coding Potential Calculator algorithm (CPC2), and txCdsPredict from UCSC were used to calculate the coding potential of lncARAT. The results from all three algorithms showed that lncARAT does not possess protein-coding potential. (G) qRT-PCR analysis was carried out to assess lncARAT expression after injecting Lv-lncARAT or Lv-shlncARAT into sciatic nerves at 4 and 7 dpi. **p < 0.01. [file 10020_2022_501_MOESM1_ESM.jpg]

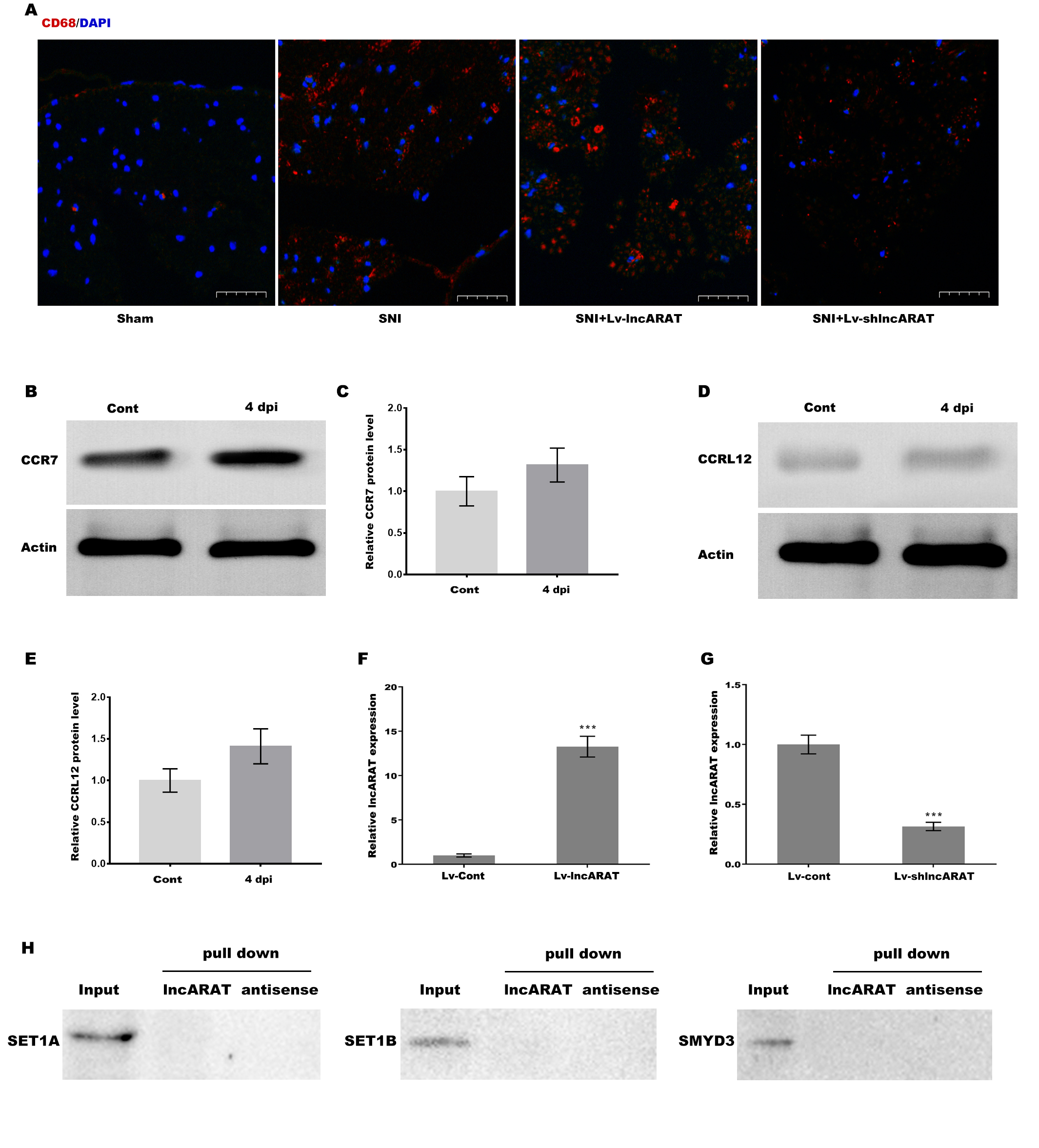

Supplement: Supplementary file 2 — Additional file 2: Figure S2. LncARAT promoted infiltration of macrophages into injury site. (A) Immunofluorescence assay of CD68 (red) and DAPI (blue) in injured nerves after lncARAT overexpression or knockdown. Scale bar, 50 µm. (B) qRT-PCR analysis of CCR7 expression in injured nerves at 4 dpi. Western blot (C) and quantitative (D) analysis of CCR7 expression in injured nerves at 4 dpi. (E) Western blot analysis of CCRL12 expression in injured nerves at 4 dpi. qRT-PCR analysis was carried out to assess lncARAT expression after treatment with Lv-lncARAT (F) or Lv-shlncARAT (G) in primary SCs. (H) RNA pull down assay followed by western blot analysis of SET1A, SET1B, and SMYD3.***p < 0.001. [file 10020_2022_501_MOESM2_ESM.jpg]

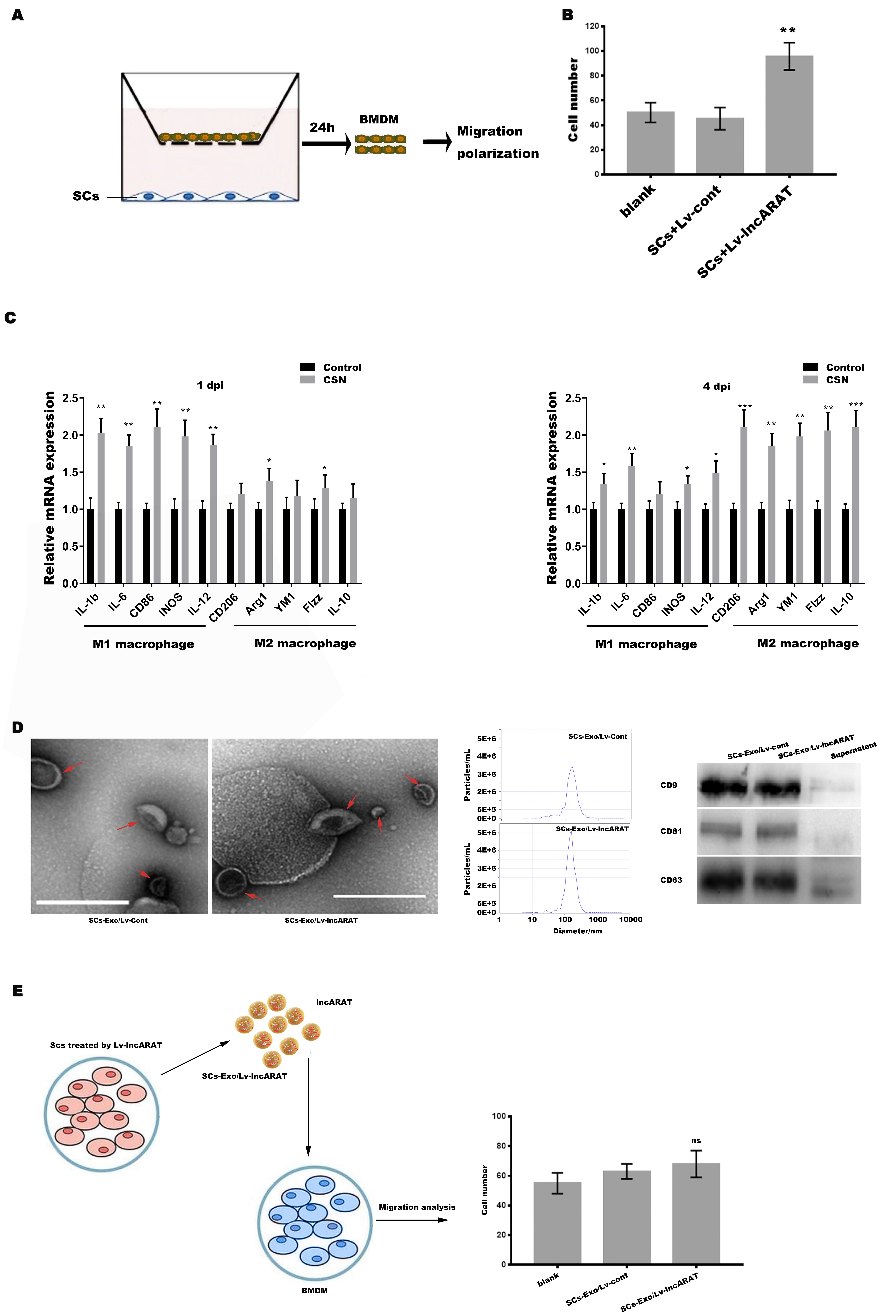

Supplement: Supplementary file 3 — Additional file 3: Figure S3. SCs-Exo promoted macrophage M2 polarization. (A) Schematic presentation of an in vitro co-culture system for assessing the role of SCs in regulating BMDM migration and polarization. (B) Transwell migration assay of BMDM co-cultured with SCs with or without lncARAT overexpression. (C) qRT-PCR analysis was carried out to assess the expression of macrophage activation markers in crushed sciatic nerves (CSN) at 1 dpi and 4 dpi. (D) Exosomes (indicated by red arrows) were isolated from SCs-Exo/Lv-cont and SCs-Exo/Lv-lncARAT and analyzed through TEM, ZetaView® Nanoparticle-tracking analysis (NTA) equipment, and western blot analysis of exosomal markers (CD9, CD81, and CD63). Scale bar, 500 nm. (E) Schematic presentation for assessing BMDM migration. In brief, SCs were treated with Lv-lncARAT to overexpress lncARAT and then SCs-Exo/Lv-lncARAT was collected. Transwell migration assay was carried out to assess BMDM migration after SCs-Exo/Lv-lncARAT treatment. ns, no significant. [file 10020_2022_501_MOESM3_ESM.jpg]

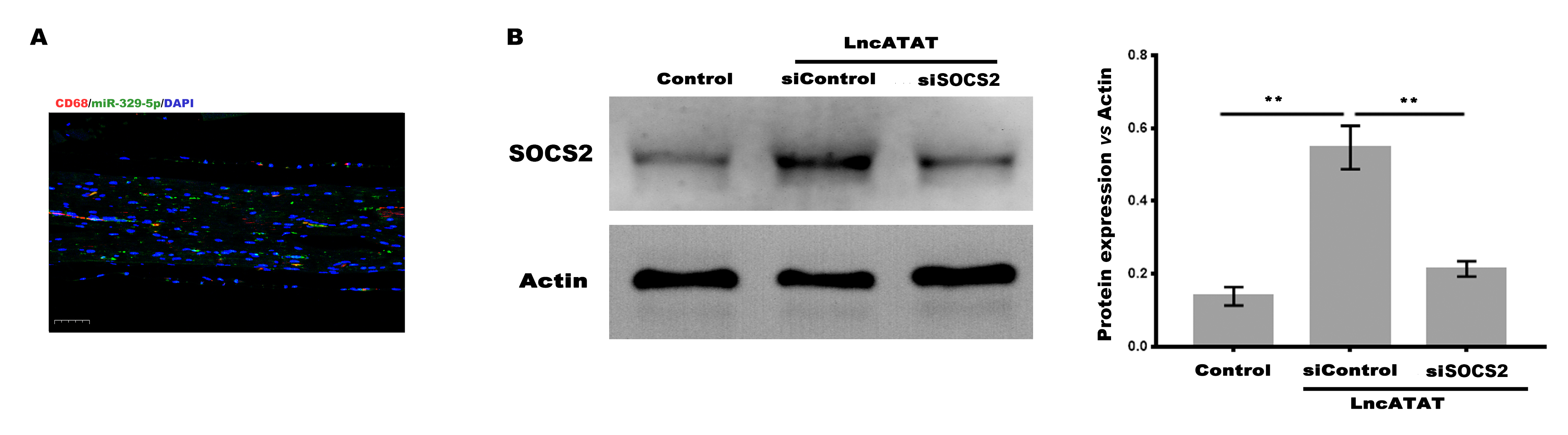

Supplement: Supplementary file 4 — Additional file 4: Figure S4. lncARAT upregulated SOCS2 expression. (A) Immunofluorescence analysis of CD68 (red) and miRNA-329-5p (green) to assess miRNA-329-5p expression in infiltrating macrophages following injury. Scale bar, 50 µm. (B) Western blot and quantitative analysis of SOCS2 expression in U937 cell after lncARAT overexpression in the presence or absence of siSOCS2. **p < 0.01. [file 10020_2022_501_MOESM4_ESM.jpg]

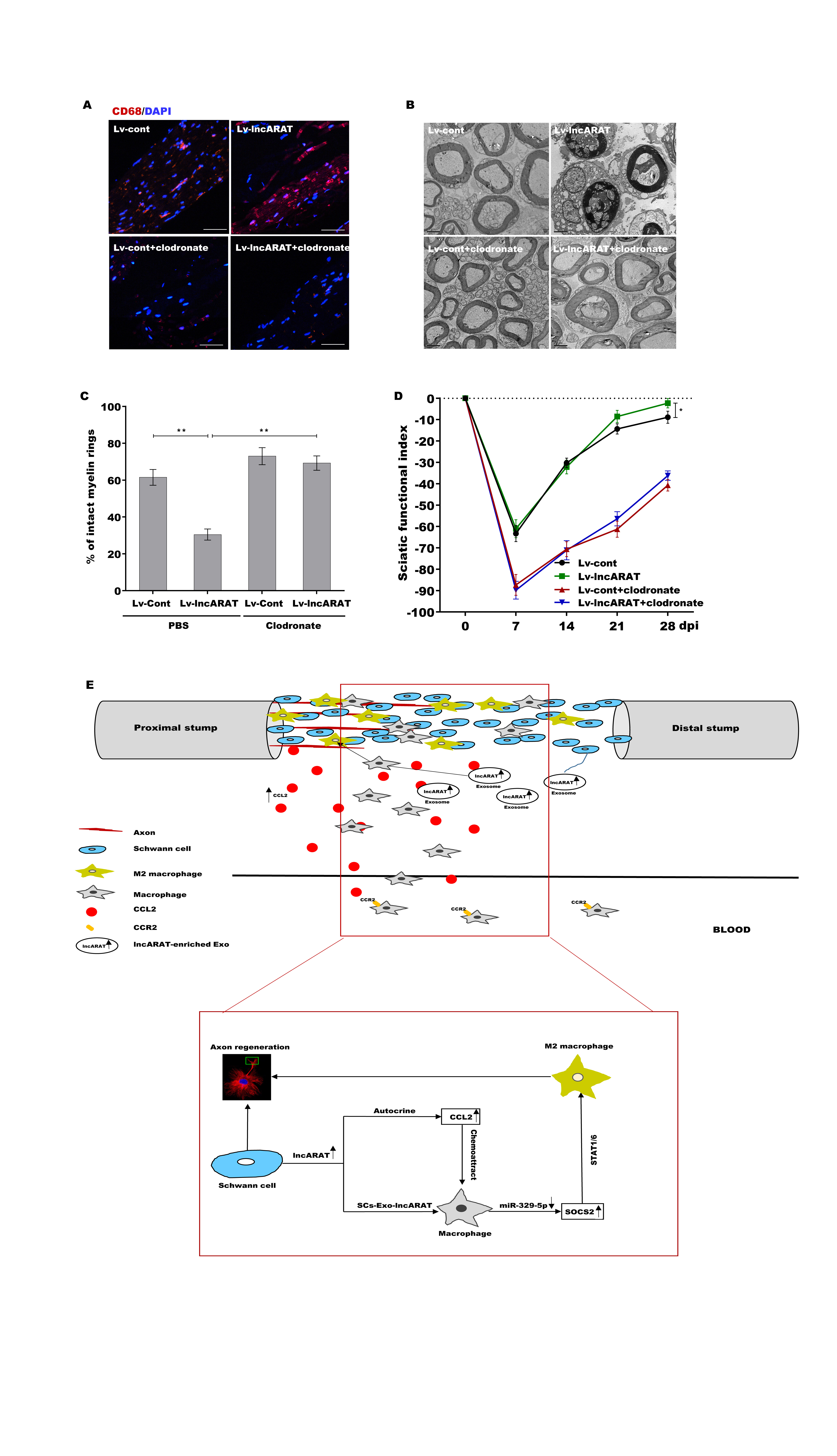

Supplement: Supplementary file 5 — Additional file 5: Figure S5. lncARAT facilitated axonal regeneration and functional recovery through interacting with macrophages. (A) An in vivo macrophage-exhausted model was established by injecting clodronate in the presence of Lv-lncARAT and damaged the sciatic nerves after 4 days, and then immunofluorescence analysis of CD68 (red) and DAPI (blue) in injured nerves was carried out to assess macrophage infiltration. Scale bar, 50 µm. (B and C) An in vivo macrophage-exhausted model was established by injecting clodronate in the presence of Lv-lncARAT and damaged the sciatic nerves after 4 days, and then electron micrographs showed the function of lncARAT and clodronate on accelerating early demyelination. Scale bar, 2 µm. (D) Plot of SFI obtained with walking track analysis after sciatic nerve crush after lncARAT overexpression in the presence or absence of clodronate (n = 5 per group). (E) Schematic presentation of the interaction between SCs and macrophages in axonal regeneration. In brief, lncRNA expression was increased in SCs and SCs-derived exosomes after crushed sciatic nerves. Upregulated lncARAT epigenetically activated CCL2 expression by recruiting KMT2A to CCL2 promoter. CCL2 facilitated the infiltration of macrophages into the injured nerves. Meanwhile, lncARAT-enriched exosomes were released from SCs and incorporated into macrophages. LncARAT functioned as an endogenous sponge to adsorb miRNA-329-5p in macrophage, resulting in an increased SOCS 2 expression, which facilitated macrophage M2 polarization through a STAT 1/6-dependent pathway, thus promoted axonal regeneration. [file 10020_2022_501_MOESM5_ESM.jpg]
